# Ruthenium-catalyzed C–H activation of thioxanthones

**DOI:** 10.3762/bjoc.11.49

**Published:** 2015-04-02

**Authors:** Danny Wagner, Stefan Bräse

**Affiliations:** 1 Department of Chemistry, Karlsruhe Institute of Technology, Fritz-Haber-Weg 6, 76131 Karlsruhe, Germany; 2 Institute of Toxicology and Genetics, Karlsruhe Institute of Technology, Campus North, Hermann-von-Helmholtz-Platz 1, 76344 Eggenstein-Leopoldshafen, Germany

**Keywords:** C–H activation, metal catalysis, thioxanthones

## Abstract

Thioxanthones – being readily available in one step from thiosalicylic acid and arenes – were used in ruthenium-catalyzed C–H-activation reaction to produce 1-mono- or 1,8-disubstituted thioxanthones in good to excellent yields. Scope and limitation of this reaction are presented.

## Introduction

**Figure 1:**
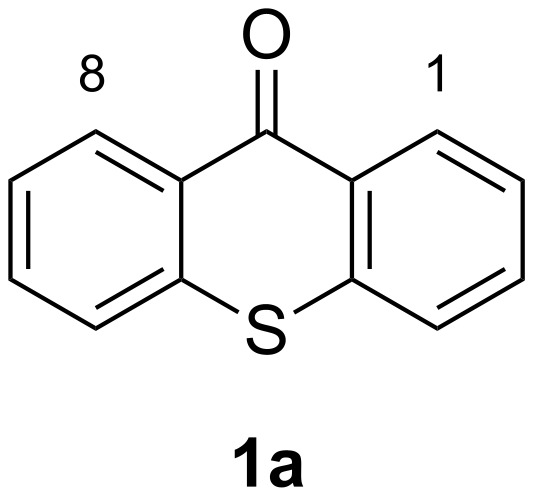
Thioxanthone (**1a**).

Thioxanthones (Figure 1) belong as a unique member to the large group of benzoannelated heterocycles [1]. They have found extensive use in biomedical applications (drugs and other bioactive compounds [2–5]) and material sciences, e.g., as photosensitizers (e.g., isopropylthioxanthone or diethylthioxanthone) [6–8] or as ligands [9–10]. Despite the widespread occurrences, there are only few modular syntheses reported so far and photosensitizing materials are often used as undefined mixtures. In addition, functionalization reactions for thioxanthones, such as C–C-bond formations [11–12], are underdeveloped [13]. For example, there are only a handful of 1,8-dialkyl/aryl-functionalized thioxanthones known [14–16], while more than 500 1-substituted thioxanthones are reported according to Scifinder. In contrast, xanthone chemistry aiming at a high degree of substitution seems to be well explored [17–18].

This fact motivated us to extend existing methods [14–15] for the synthesis of substituted thioxanthones. We were intrigued by the fact that carbonyl-substituted arenes can undergo a smooth C–H activation and alkylation in the presence of metal catalysts [19] (for general reviews see [20–21]). However, there are only few examples [14–15] with sulfur-containing heterocycles as in general sulfur inhibits the catalytic activity of many transition metal catalysts [22].

## Results and Discussion

### Synthesis of functionalized thioxanthones

The required thioxanthones **1** were prepared using standard procedures [1,23]. For certain examples, optimizations of the standard protocol were required (Table 1 and Supporting Information File 1).

**Table 1:** Synthesis of substituted thioxanthones from thiosalicylic acid.^a^

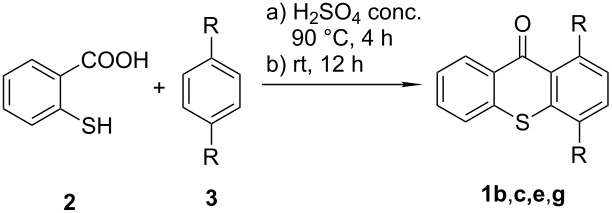			

Entry	Arene **3**	Thioxanthone **1**	Yield^b^

1	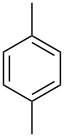 **3b**	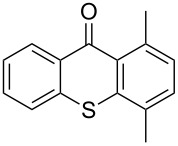 **1b**	57
2	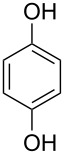 **3c**	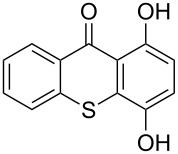 **1c**	9
3	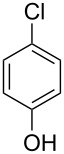 **3e**	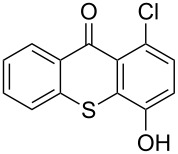 **1e**	87
4	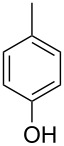 **3h**	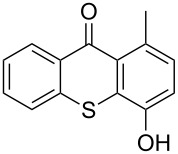 **1g**	25

^a^For conditions see Supporting Information File 1; ^b^isolated yields.

**Scheme 1:**
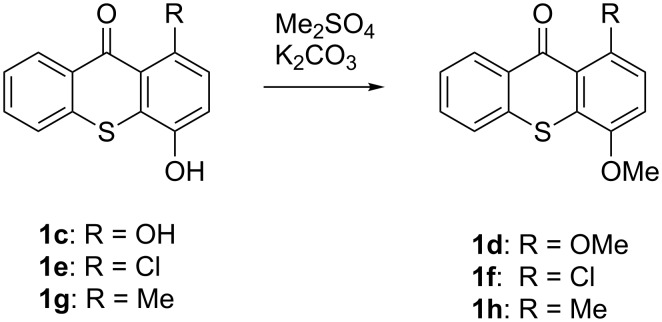
Route to methoxyarenes **1d**, **1f**, and **1h**.

In case of methoxyarenes this method was not successful due to a partial ether cleavage catalyzed by hot sulfuric acid. In this case, methylation (Me_2_SO_4_, K_2_CO_3_) of the hydroxythioxanthones **1c**, **1e** and **1g** provided the required methyl ethers **1d**, **1f** and **1h**, respectively in good yields (80, 85 and 95%), Scheme 1.

### Ru-catalyzed C–H activation

**Scheme 2:**
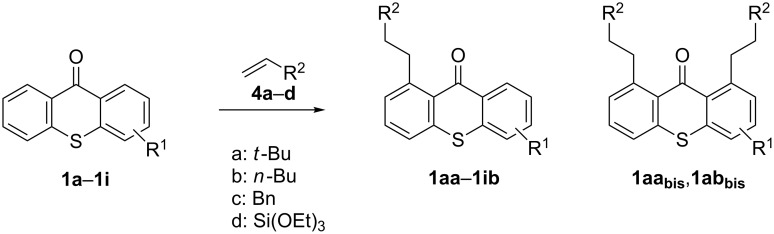
Ru-catalyzed C–H activation of thioxanthones. Conditions: 6 mol % RuH_2_(CO)(PPh_3_)_3_, toluene, 135 °C, 12 h.

Following the precedence for other carbonyl compounds, we used the protocol of Murai et al. [19] to investigate the use of thioxanthones in this C–H-alkylation reaction (Scheme 2). For recent examples and reviews, also for related rhodium-catalyzed systems, see [24–38].

It should be noted that already in the pioneering work there are also examples using sulfur heterocycles such as thiophene derivatives [19]. Gratifyingly, the reaction of dimethyl-substituted thioxanthone **1b** with the model olefin neohexene (3,3-dimethyl-1-butene, **4a**) was successful and the product **1ba** was obtained in 65% yield (Table 2, entry 4). This and all the products obtained exhibit exclusively *n*-alkylation – branched alkyl chains originating from addition at the 2-position were not found. Other olefins like 1-hexene (**4b**) or 3-phenylpropene (**4c**) also worked smoothly (Table 2, entries 5 and 6). In addition, the silyl-substituted olefin **4d** was also successfully used in this reaction (Table 2, entry 7). The product **1bd** is formed in good yield, however, it is prone to hydrolysis thus the isolated yield of the pure product was rather low. In contrast to literature precedence for other carbonyl compounds [19], other olefins (styrene, vinyl/allyl ethers, perfluoroalkylethenes) failed since they polymerize during the reaction.

**Table 2:** C–H activation.

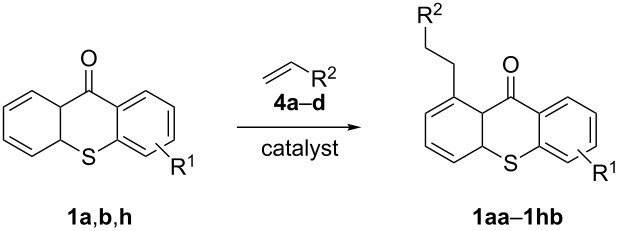				

Entry	Thioxanthone	Alkene (equiv)	Product	Yield [%]^a^

1	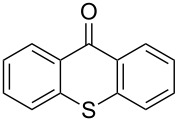 **1a**	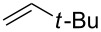 **4a** (1.2 equiv)	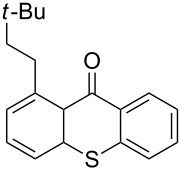 **1aa**	47
			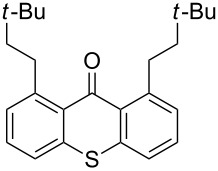 **1aa_bis_**	20
2	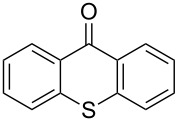 **1a**	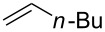 **4b** (6 equiv)	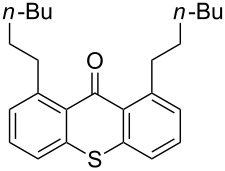 **1ab_bis_**	43
3	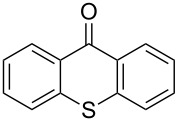 **1a**	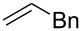 **4c** (1.2 equiv)	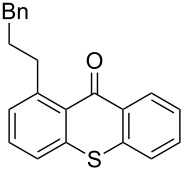 **1ac**	7
4	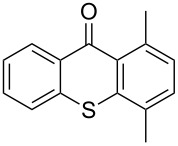 **1b**	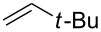 **4a** (3 equiv)	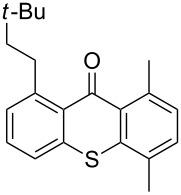 **1ba**	65
5	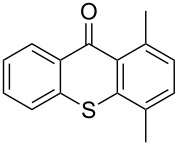 **1b**	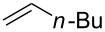 **4b** (3 equiv)	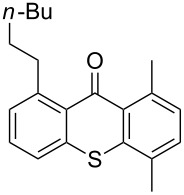 **1bb**	66
6	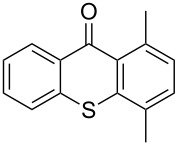 **1b**	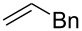 **4c** (3 equiv)	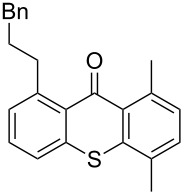 **1bc**	27
7	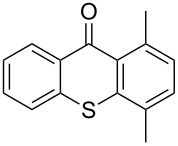 **1b**	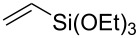 **4d** (3 equiv)	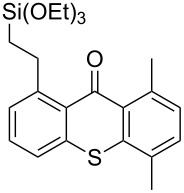 **1bd**	55^b^
8	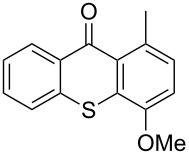 **1h**	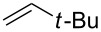 **4a** (3 equiv)	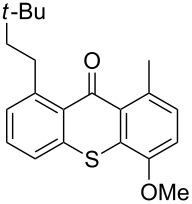 **1ha**	83
9	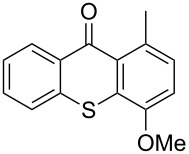 **1h**	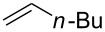 **4b** (3 equiv)	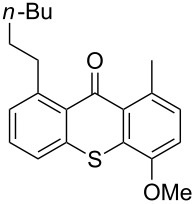 **1hb**	65

^a^Isolated yields; ^b^crude yield close to quantitative, but product prone to hydrolysis.

Extension of the unsymmetrical heterocycle system **1b** to the unsubstituted thioxanthone (**1a**) was also successful: Depending on the amount of alkene, mono- (Table 2, entry 3) or 1,8-disubstituted thioxanthones such as **1aa_bis_** or **1ab_bis_** were isolated (see Table 2, entries 1 and 2). In addition, other thioxanthones such as **1h** are also suitable substrates (Table 2, entries 8 and 9).

However, other thioxanthones such as a phenanthrene-annelated thioxanthone (not shown) failed to give the desired products due to insolubility of the starting materials.

## Conclusion

We have presented a C–H-activation route towards the preparation of functionalized thioxanthones. Despite the fact that mono- and disubstituted thioxanthones can be found starting from the parent system, the selectivity can be controlled using different stoichiometries. It should be noted that alkoxy and silyl functionalities are tolerated in the reaction.

## Experimental

The catalyst RuH_2_(CO)(PPh_3_)_3_ was prepared according to literature [19] and stored under Argon with exclusion of water.

### General procedure for C–H activation

In a sealed Schlenk pressure tube, 1.00 mmol of the thioxanthone, 0.060 mmol (55 mg) RuH_2_(CO)(PPh_3_)_3_, 1.2 to 6 mmol of the olefin and 2 mL toluene were stirred and heated to 135 °C for 12 h. After cooling, the solvent was evaporated under reduced pressure and the residue submitted to column chromatography on silica gel using cyclohexane/ethyl acetate as eluent.

## Supporting Information

File 1 Characterization data and spectra for compounds **2** and **3**.
