# Supplementary material for: Ruthenium-catalyzed C–H activation of thioxanthones
Source: Beilstein J Org Chem. 2015 Apr 2;11:431–6. doi: 10.3762/bjoc.11.49 (PMC4419513; doi:10.3762/bjoc.11.49)
Supplement: File 1 — Characterization data and spectra for compounds 2 and 3. [file Beilstein_J_Org_Chem-11-431-s001.pdf]

Supporting Information

for

Ruthenium-catalyzed C–H activation of thioxanthones

Danny Wagner[1] and Stefan Bräse[1,2]*

Address: [1]Department of Chemistry, Karlsruhe Institute of Technology, Fritz-Haber-Weg 6, 76131 Karlsruhe, Germany and [2]Institute of Toxicology and Genetics, Karlsruhe Institute of Technology, Campus North, Hermann-von-Helmholtz-Platz 1, 76344 Eggenstein-Leopoldshafen, Germany

Email: Stefan Bräse - braese@kit.edu

*Corresponding author

Characterization data and spectra for compounds 2 and 3.

Table of contents:

1 Materials and methods S2

2 Analytical data S2

1 Materials and methods

^{1}H NMR spectra were recorded on a BRUKER Avance 400 (400 MHz) device at room temperature. Chemical shifts are expressed in parts per million (ppm, δ), downfield from tetramethylsilane (TMS) and referenced to residual solvent resonance (chloroform, 7.26 ppm) as internal standard. All coupling constants are absolute values and *J* values are expressed in Hertz (Hz). The spectra were analyzed according to first order and the descriptions of signals include: s = singlet, d = doublet, dd = doublet of doublets, t = triplet, q = quartet, m = multiplet. **^{13}C NMR** spectra were recorded on a BRUKER Avance 400 (100 MHz) device at room temperature. Chemical shifts are expressed in parts per million (ppm, δ), downfield from tetramethylsilane (TMS) and referenced to residual solvent resonance ($CDCl_3$, 77.0 ppm) as internal standard. The signal structure was analyzed by DEPT and is described as follows: + = primary or tertiary C-atom (positive DEPT signal), – = secondary C-atom (negative DEPT signal), and C_q = quaternary C-atom (no DEPT signal).
EIMS (electron ionization mass spectrometry) was performed using a *Finnigan* MAT 95 (70 eV). The molecular fragments are quoted as relation between mass and charge (*m*/*z*), the intensities as percentage values relative to the intensity of the base signal (100%). The abbreviation $[M]^+$ refers to the molecular ion.
IR (infrared spectroscopy) data were recorded on a *Bruker* Alpha FTIR spectrometer and are reported as follows: frequency of absorption (cm^{-1}), intensity of absorption (vs = very strong (81–100%), s = strong (61–80%), m = medium (41–60%), w = weak (21–40%), vw = very weak (1–20%)).
Reactions were monitored by silica gel coated aluminium plates (*Merck*, silica gel 60, F_{254}). Detection was performed by examination under UV light (254 nm). Solvents, reagents and chemicals were purchased from *Sigma-Aldrich and ABCR*. Toluene was distilled from sodium prior to use. All reactions were executed under argon atmosphere using oven-dried glassware. All other chemicals were used as purchased unless stated otherwise.

2 Analytical data

8-(3,3-Dimethylbutyl)-1,4-dimethyl-9H-thioxanthen-9-one (1ba)

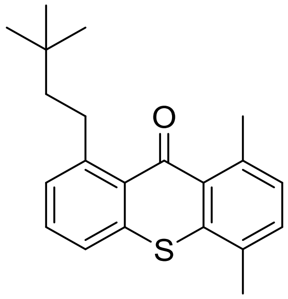

65% yield, clear oil.
^{1}H NMR (400 MHz, $CDCl_3$): δ = 1.04 (s, 9 H, C(CH_3)$_3$), 1.54 (dt, 2 H, CH_2), 2.47 (s, 3 H, CH_3 Thiox.), 2.72 (s, 3 H, CH_3 Thiox.), 3.15 (dt, 2 H, CH_2), 7.15 (d, 1 H, $H_{ar.}$), 7.23 – 7.28 (m, 2 H, $H_{ar.}$), 7.37 -7.44 (m, 2 H, $H_{ar.}$) – ^{13}C NMR (100 MHz, $CDCl_3$): δ = 19.5 (+, CH$_3$ Thiox.), 21.8 (+, CH$_3$ Thiox.), 29.4 (+, C(CH)$_3$), 29.8 (-, CH$_2$, CH$_2$), 30.9 ($C_{q.}$, C(CH$_3$)$_3$), 46.8 (-, CH$_2$, CH$_2$), 123.6 (+, CH, 5-CH$_{TX}$), 128.9 (+, CH, 7-CH$_{TX}$), 129.1 (+, CH, 2-CH$_{TX}$), 130.4 (+, CH, 6-CH$_{TX}$), 131.1 (C_q., C_{TX}), 131.3 ($C_{q.}$, C_{TX}), 131.5 (+, CH, 3-CH$_{TX}$), 132.0 ($C_{q.}$, C_{TX}), 135.6 ($C_{q.}$, C_{TX}), 136.2 ($C_{q.}$, C_{TX}), 137.8 ($C_{q.}$, C_{TX}), 146.2 ($C_{q.}$, CCH$_2$), 187.8 ($C_{q.}$, CO) ppm. – MS (70 eV, EI), *m/z* (%): 324 (95) [M$^+$], 267 (100) [$C_{17}H_{15}OS^+$]. – HRMS ($C_{21}H_{24}OS$): calc. 324.1542; found 324.1542 – IR (ATR): $\tilde{\upsilon}$ (cm^{-1}) = 2945 (w), 1643 (m) (CO), 1564 (w), 1445 (w), 1390 (vw), 1378 (w), 1363 (w), 1292 (w), 1244 (w), 1207 (w), 1179 (vw), 1150 (vw), 1017 (vw), 953 (vw), 860 (w), 826 (w), 805 (w), 783 (m), 742 (w), 726 (vw), 707 (vw), 686 (w), 617 (vw), 556 (w), 495(vw), 447 (vw). — EA: calc. for $C_{21}H_{24}OS$: C 77.73, H 7.46, S 9.88; found C 78.06, H 7.69, S 9.61.

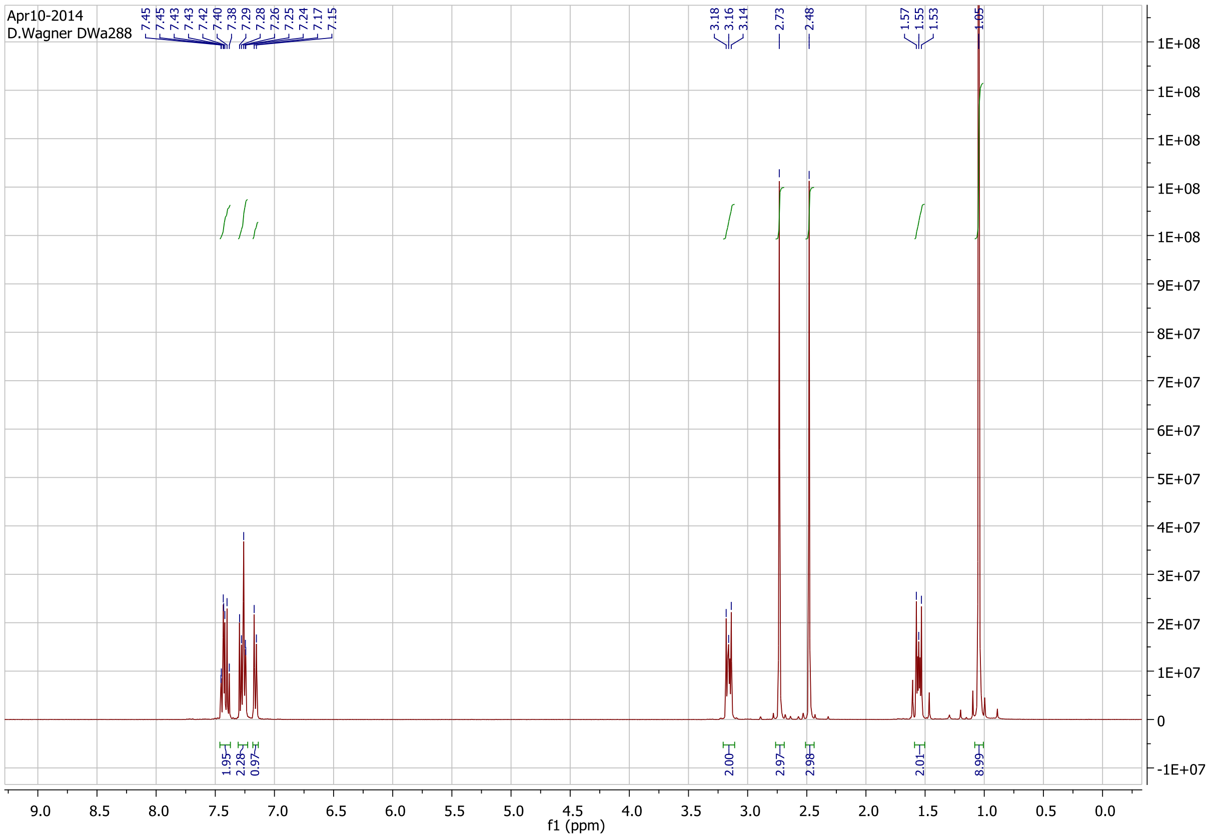
Apr10-2014
D.Wagner DWa288
7.45 7.45 7.43 7.43 7.42 7.40 7.38 7.29 7.28 7.26 7.25 7.24 7.17 7.15
3.18 3.16 3.14
2.73
2.48
1.57 1.55 1.53
1.05
1.95 2.28 0.97
2.00
2.97
2.98
2.01
8.99
f1 (ppm)

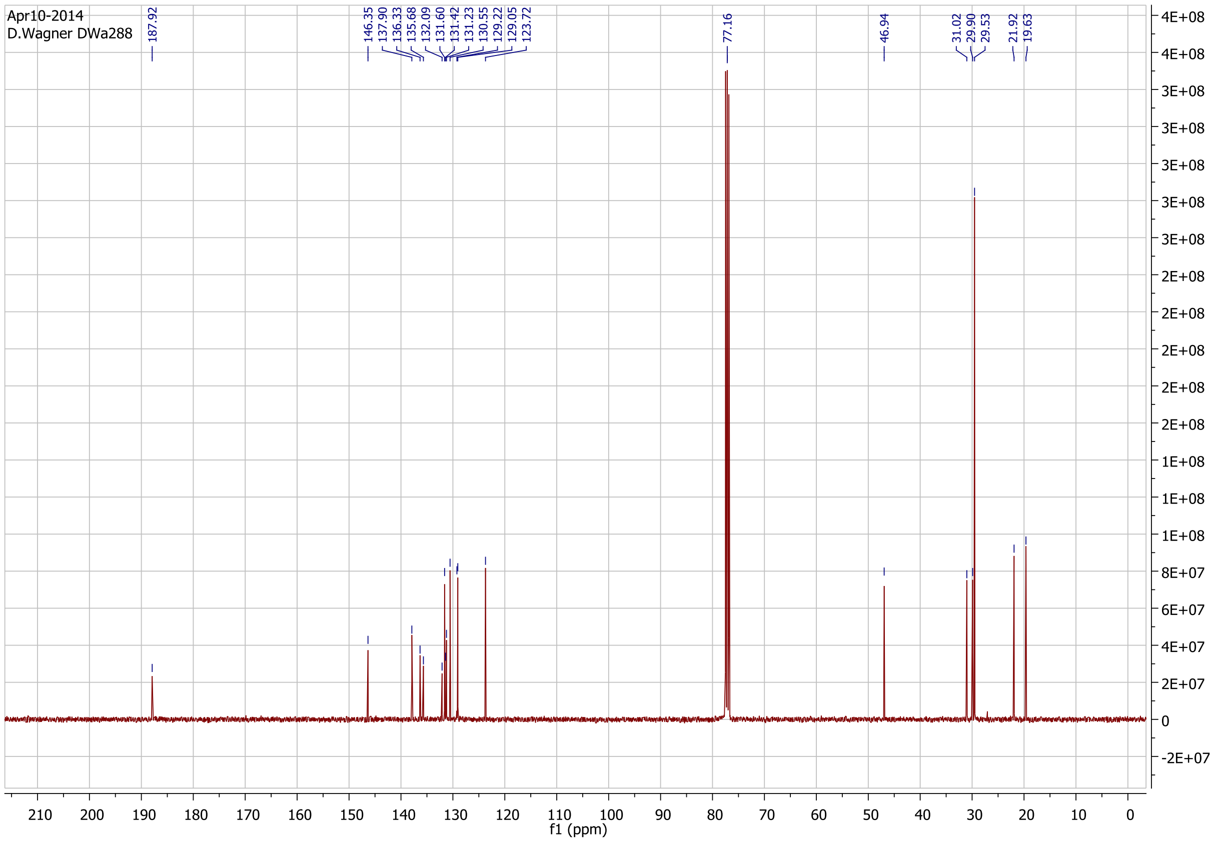
Apr10-2014
D.Wagner DWa288
187.92
146.35 137.90 136.33 135.68 132.09 131.60 131.42 131.23 130.55 129.22 129.05 123.72
77.16
46.94
31.02 29.90 29.53
21.92 19.63
f1 (ppm)

1,4-Dimethyl-8-(3-phenylpropyl)-9H-thioxanthen-9-one (**1bc**)

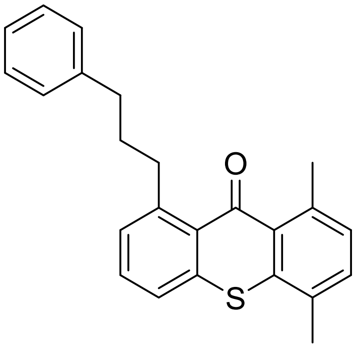

27% yield, clear oil.

^{1}H NMR (400 MHz, $CDCl_3$): δ = 2.04 (dq, 2 H, $CH_2CH_2CH_2$), 2.48 (s, 3 H, CH_3 Thiox.), 2.69 (s, 3 H, CH_3 Thiox.), 2.77 (t, 2 H, $CH_2C_6H_5$), 3.22 (t, 2 H, CH_2 Thiox.), 7.15 – 7.25 (m, 5 H, $H_{ar.}$), 7.28 -7.47 (m, 5 H, $H_{ar.}$) – ^{13}C NMR (100 MHz, $CDCl_3$): δ = 19.62 (+, CH_3 Thiox.), 22.07 (+, CH_3 Thiox.), 33.7 (-, CH_2, $CH_2CH_2CH_2$), 34.2 (-, CH_2, CH_2 Thiox.), 36.1 (-, CH2, CH_2Ph), 124.0 (+, CH, 5-CH_{TX}) 125.8 (+, CH, 4'-CH), 128.4 (+, CH, 2'-CH, 6'-CH), 128.6 (+, CH, 3'-CH, 5'-CH), 129.08 (+, CH, 2-CH_{TX}), 129.1 (+, CH, 7-CH_{TX}), 130.5 (+, CH, 3-CH_{TX}), 131.2 ($C_{q.}$, C_{TX}), 131.4 ($C_{q.}$, C_{TX}), 131.7 (+, CH, 6-CH_{TX}), 132.0 ($C_{q.}$, C_{TX}), 135.7 ($C_{q.}$, C_{TX}), 136.5 ($C_{q.}$, C_{TX}), 138.0 ($C_{q.}$, C_{TX}), 142.5 ($C_{q.}$, C_{TX}), 145.0 ($C_{q.}$, C_{Ph}), 187.9 ($C_{q.}$, CO) ppm. – MS (70 eV, EI), *m/z* (%): 358 (85) [M^+], 267 (65) [$C_{17}H_{15}OS^+$], 254 (100) [$C_{16}H_{14}OS^+$]. – HRMS ($C_{24}H_{22}OS$): calcd. 358.1386; found 358.1388 – IR (ATR): $\tilde{\upsilon}$ (cm^{-1}) = 2922 (vw), 2852 (vw), 1648 (w) (CO), 1562 (w), 1494 (vw), 1444 (w), 1378 (vw), 1281 (w), 1254 (vw), 1204 (vw), 1177 (vw), 1083 (vw), 1032 (vw), 958 (vw), 905 (vw), 862 (vw), 814 (w), 804 (w), 782 (w), 749 (w), 727 (w), 697 (w), 621 (vw), 587 (vw), 554 (vw), 483 (vw), 445 (vw). — EA: calcd. for $C_{24}H_{22}OS$: C 80.41, H 6.19, S 8.94; found C 80.52, H 6.39, S 8.61.

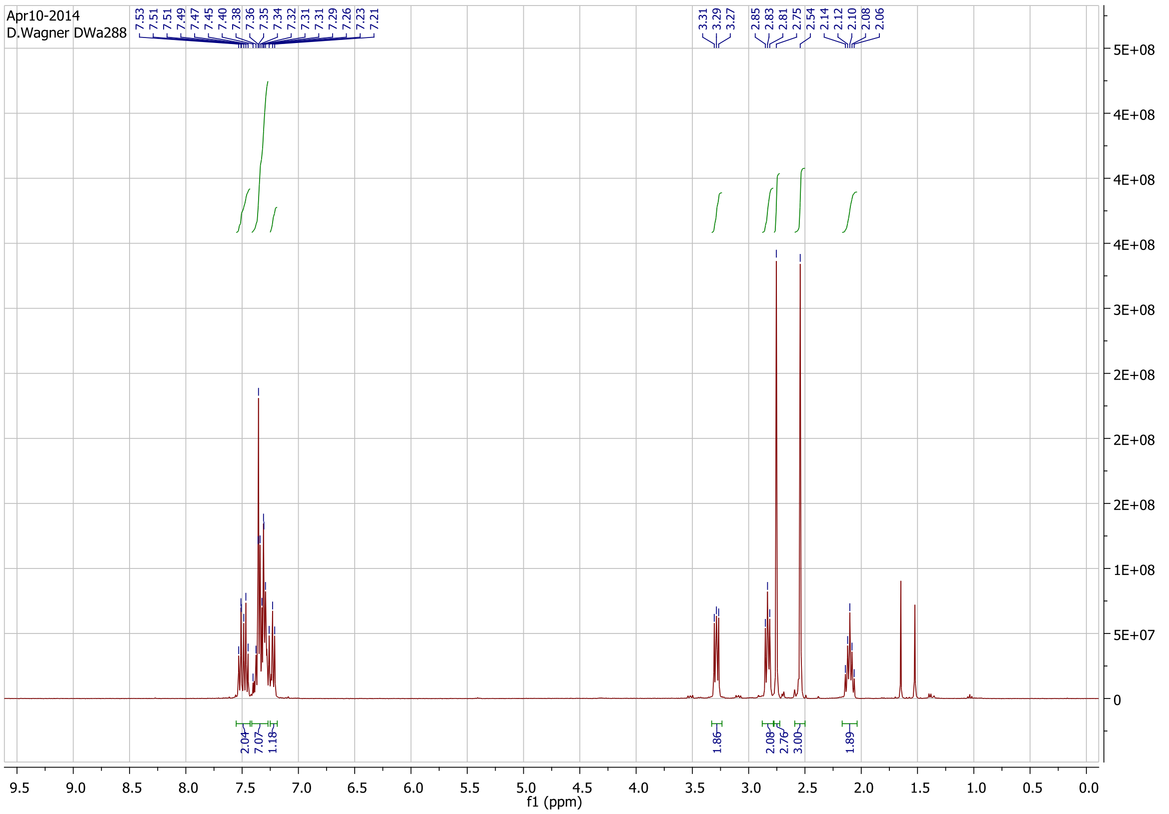

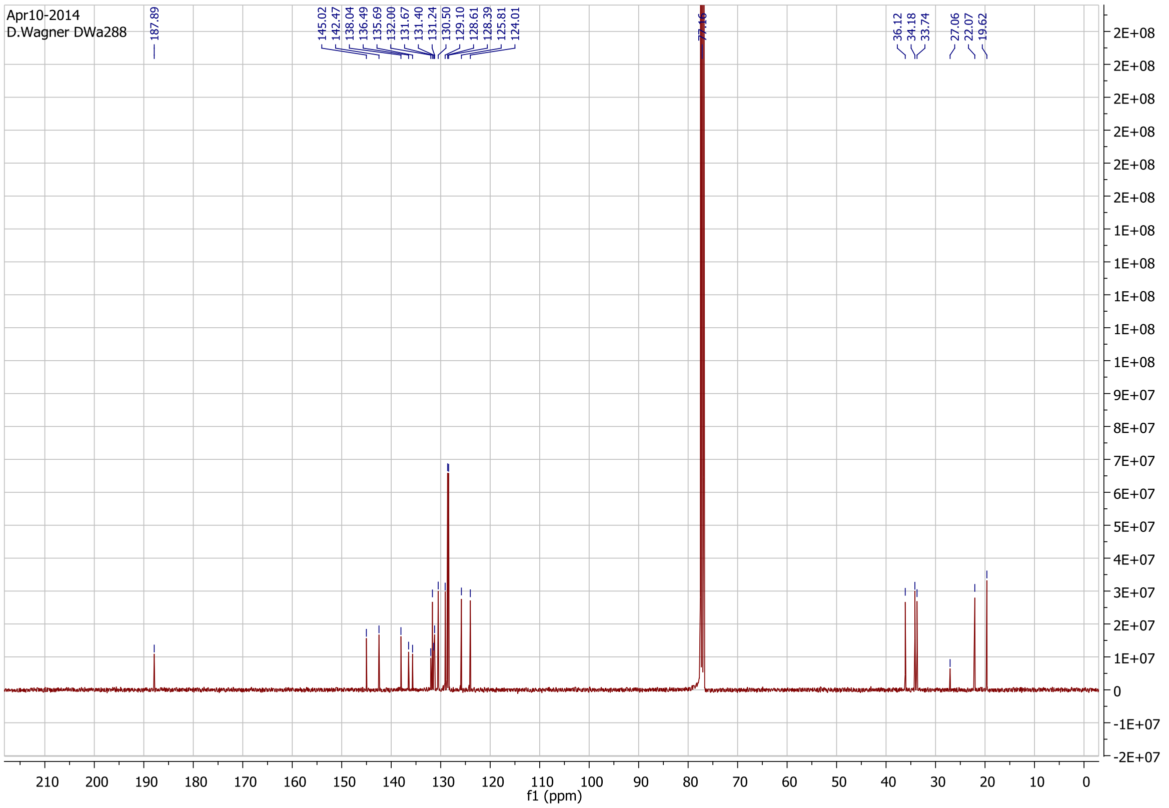

8-Hexyl-1,4-dimethyl-9H-thioxanthen-9-one (**1bb**)

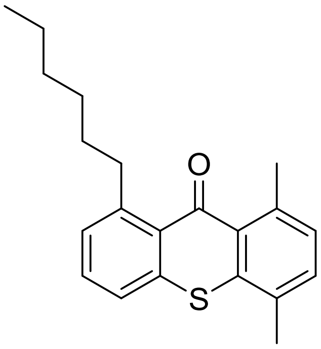

66% yield, clear oil.

^{1}H NMR (400 MHz, $CDCl_3$): δ = 0.88 (t, 3 H, CH_2CH_3), 1.21-1.46 (m, 6 H, $CH_2CH_2CH_2$), 1.64 (m, 2 H, CH_2), 2.45 (s, 3 H, CH_3 Thiox.), 2.68 (s, 3 H, CH_3 Thiox.), 3.14 (t, 2 H, CH_2 Thiox.), 7.13 (d, 1 H, $H_{ar.}$), 7.21 – 7.25 (m, 2 H, $H_{ar.}$), 7.35 -7.42 (m, 2 H, $H_{ar.}$) – ^{13}C NMR (100 MHz, $CDCl_3$): δ = 15.3 (+, CH_3 Hex.), 19.6 (+, CH_3 Thiox.), 21.9 (+, CH_3 Thiox.), 22.8 (-, CH_2, Hex.), 29.7 (-, CH_2, Hex.), 32.0 (-, CH_2, Hex.), 32.4 (-, CH_2, Hex.), 34.5 (-, CH_2, Hex.), 123.8 (+, CH, 5-CH_{TX}), 129.0 (+, CH, 2-CH_{TX}), 129.1 (+, CH, 7-CH_{TX}), 130.5 (+, CH, 3-CH_{TX}), 131.2 ($C_{q.}$, C_{TX}), 131.4 ($C_{q.}$, C_{TX}), 131.6 (+, CH, 6-CH_{TX}), 132.1 ($C_{q.}$, C_{TX}), 135.7 ($C_{q.}$, C_{TX}), 136.4 ($C_{q.}$, C_{TX}), 137.9 ($C_{q.}$, C_{TX}), 145.6 ($C_{q.}$, C_{TX}), 188.0 ($C_{q.}$, CO_{TX}) ppm. – MS (70 eV, EI), *m/z* (%): 324 (90) [M^+], 267 (90) [$C_{17}H_{15}OS^+$], 254 (100) [$C_{16}H_{14}OS^+$]. – HRMS ($C_{21}H_{24}OS$): calcd. 324.1542; found 324.1543 – IR (ATR): $\tilde{\nu}$ (cm^{-1}) = 3446 (vw), 3054 (vw), 2927 (m), 2856 (m), 1715 (w), 1648 (s) (CO), 1569 (m), 1445 (m), 1380 (w), 1293 (m), 1206 (w), 1173 (vw), 1100 (vw), 1015 (vw), 957 (vw), 860 (w), 817 (w), 804 (w), 782 (w), 728 (vw), 686 (vw), 555 (vw), 439 (vw), 2737 (vw).— EA: calcd. $C_{21}H_{24}OS$: C 77.73, H 7.46, S 9.88; found C 77.67, H 7.71, S 9.62.

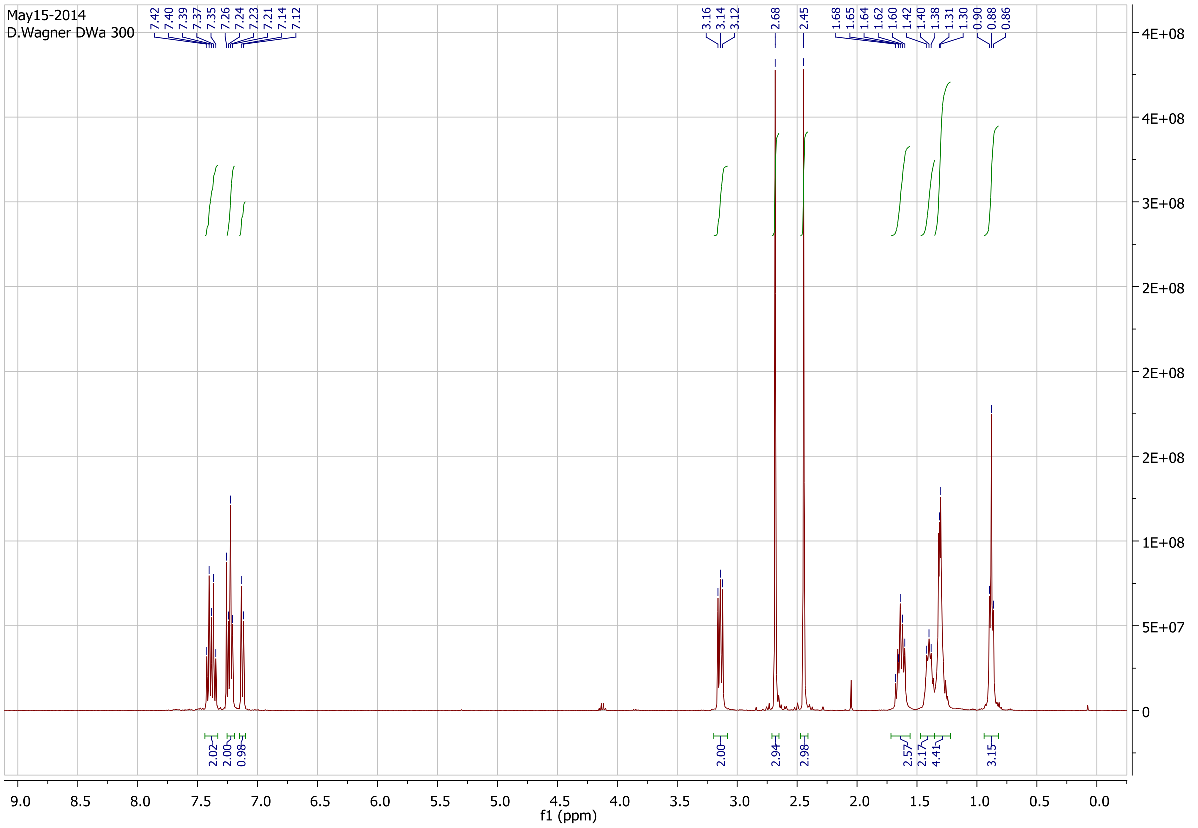
May15-2014
D.Wagner DWa 300
7.42
7.40
7.39
7.37
7.35
7.26
7.24
7.23
7.21
7.14
7.12
3.16
3.14
3.12
2.68
2.45
1.68
1.65
1.64
1.62
1.60
1.42
1.40
1.38
1.31
1.30
0.90
0.88
0.86
2.02
2.00
0.98
2.00
2.94
2.98
2.57
2.17
4.41
3.15
f1 (ppm)

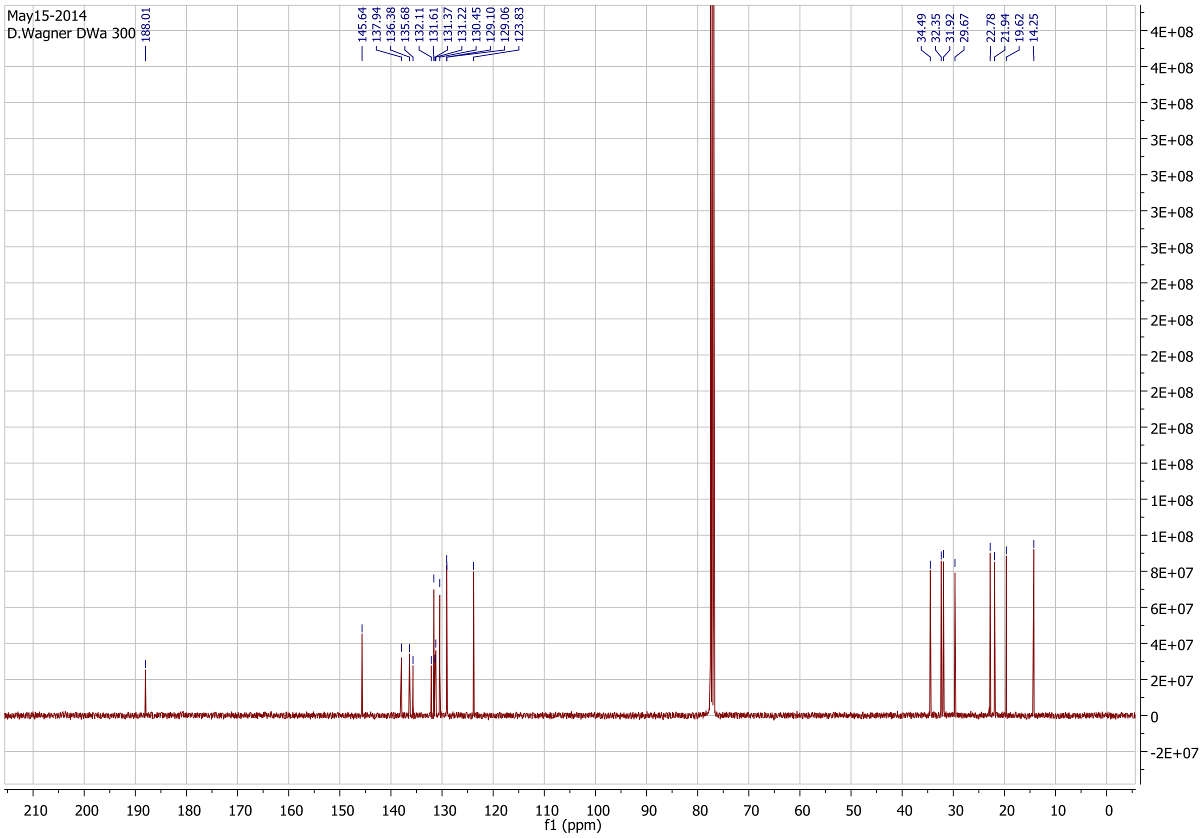
May15-2014
D.Wagner DWa 300
188.01
145.64
137.94
136.38
135.68
132.11
131.61
131.37
131.22
130.45
129.10
129.06
123.83
34.49
32.35
31.92
29.67
22.78
21.94
19.62
14.25
f1 (ppm)

1,4-Dimethyl-8-(2-(triethoxysilyl)ethyl)-9H-thioxanthen-9-one (**1bd**)

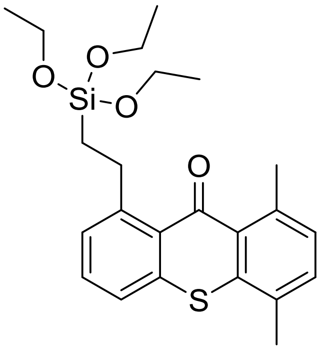

55% yield, clear oil.

^{1}H NMR (400 MHz, $CDCl_3$): δ = 1.09 – 1.11 (m, 2 H, $SiCH_2$), 1.25 (t, 9 H, $Si(OCH_2CH3)_3$), 2.43 (s, 3 H, CH_3 Thiox.), 2.67 (s, 3 H, CH_3 Thiox.), 3.16 - 3.20 (m, 2 H, CH_2), 3.86 – 3.91 (q, 6 H, $Si(OCH_2CH_3)_3$), 7.10 (d, 1 H, $H_{ar.}$), 7.20 – 7.25 (m, 2 H, $H_{ar.}$), 7.35 -7.41 (m, 2 H, $H_{ar.}$) – ^{13}C NMR (100 MHz, $CDCl_3$): δ = 13.6 (+, CH_3 Thiox.), 18.5 (-, CH_2, $Si(OCH_2CH_3)_3$), 19.6 (-, CH2, $SiCH_2$), 22.1 (-, CH_2, CH_2 Thiox.), 28.1 (+, CH_3 Thiox.), 58.5 (+, CH_3, $Si(OCH_2CH_3)_3$), 123.8 (+, CH, 5-CH_{TX}), 128.6 (+, CH, 7-CH_{TX}), 129.1 (+, CH, 2-CH_{TX}), 130.7 (+, CH, 6-CH_{TX}), 131.1 ($C_{q.}$, C_{TX}), 131.2 ($C_{q.}$, C_{TX}), 131.6 (+, CH, 3-CH_{TX}), 131.9 ($C_{q.}$, C_{TX}), 135.7 ($C_{q.}$, C_{TX}), 136.3 ($C_{q.}$, C_{TX}), 138.1 ($C_{q.}$, C_{TX}), 147.7 ($C_{q.}$, C_{TX}), 187.7 ($C_{q.}$, CO) ppm. – MS (70 eV, EI), *m/z* (%): 430 (95) $[M^+]$. – HRMS ($C_{23}H_{30}SiSO_4$): calcd. 430.1632; found 430.1634 – IR (ATR): $\tilde{\nu}$ (cm^{-1}) = 2970 (m), 2924 (w), 2889 (w), 1635 (m) (CO), 1573 (w), 1445 (m), 1380 (w), 1313 (w), 1298 (w), 1266 (vw), 1205 (vw), 1181 (m), 1071 (vs), 959 (s), 911 (w), 861 (w), 810 (w), 769 (s), 727 (w), 708 (w), 686 (w), 605 (w), 553 (w), 536 (vw), 508 (vw), 449 (w), 420 (w). — EA: calcd. $C_{23}H_{30}O_4SSi$: C 64.15, H 7.02, S 7.45; found C 63.82, H 7.56, S 6.60.

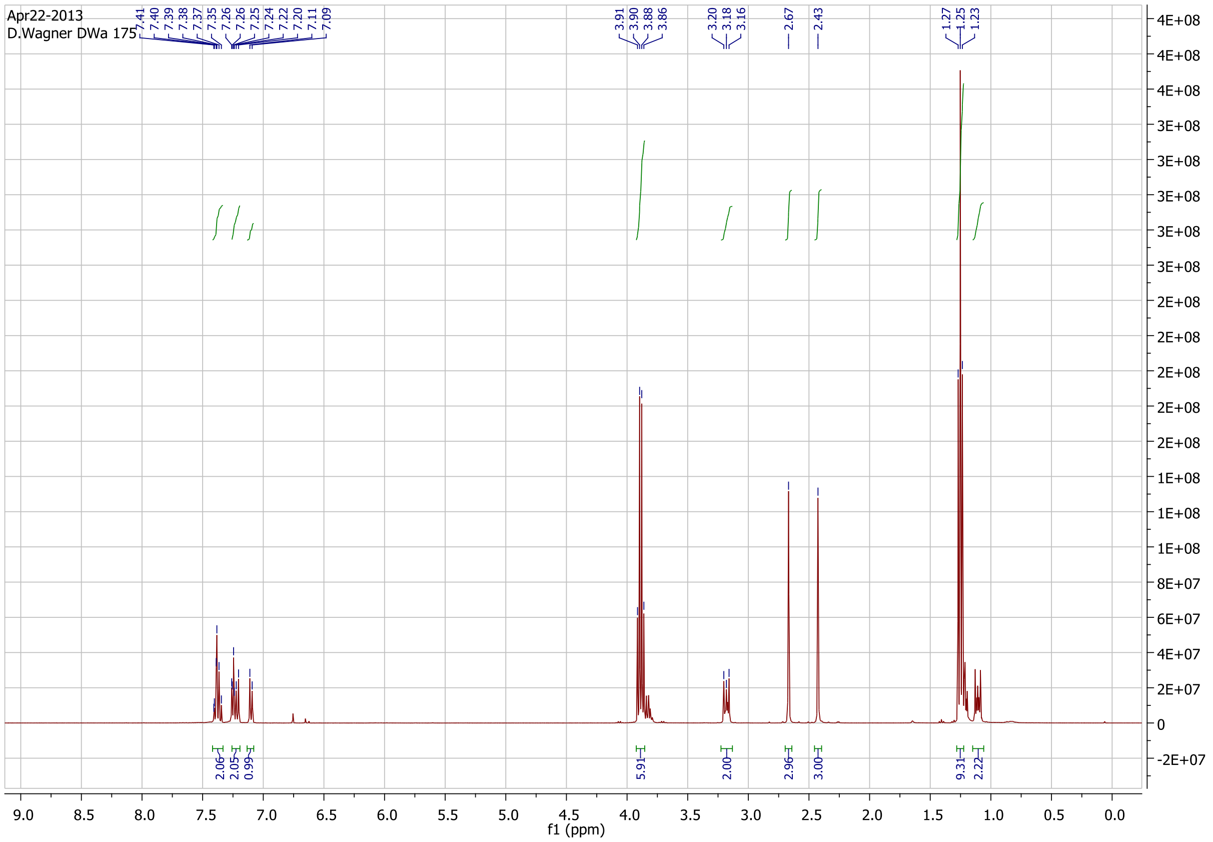

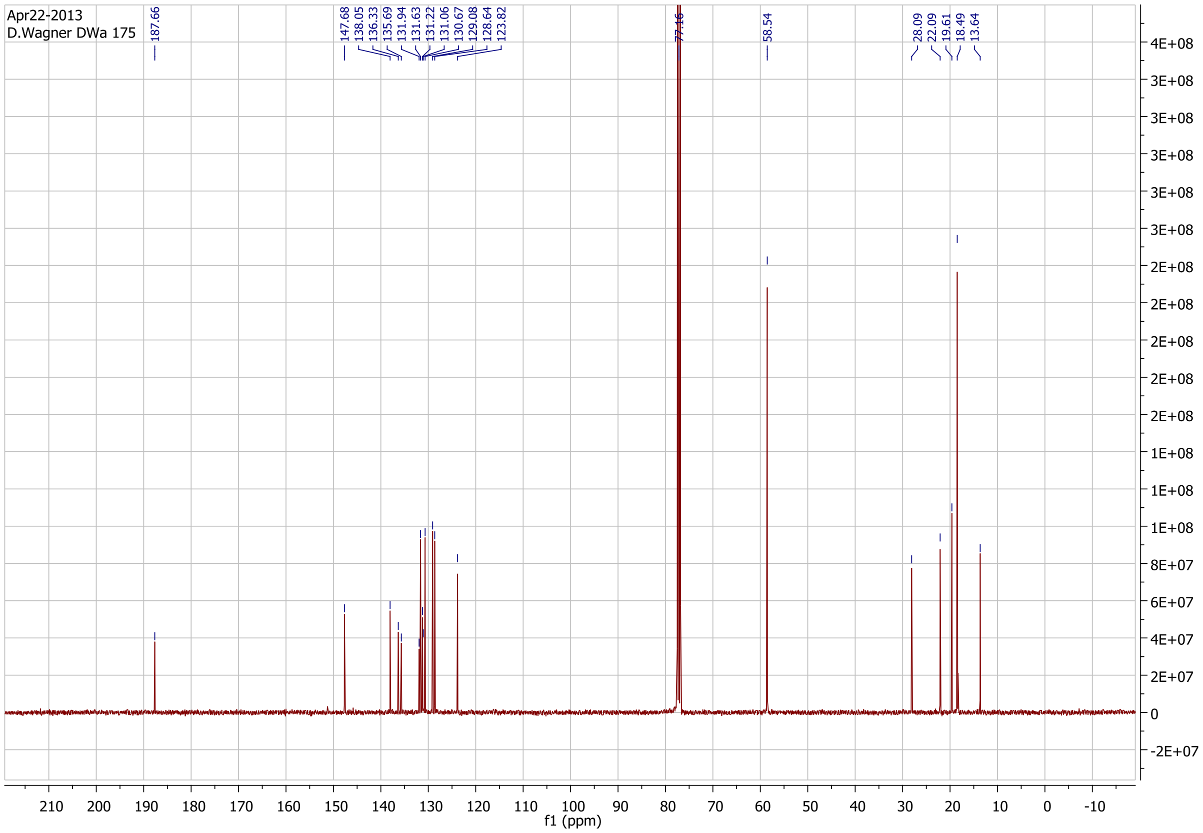

1-(3-Phenylpropyl)-9H-thioxanthen-9-one (**1ac**)

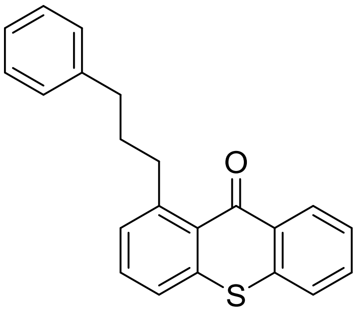

7% yield, clear oil.

^{1}H NMR (400 MHz, $CDCl_3$): δ = 2.04 (dq, 2 H, $CH_2CH_2CH_2$), 2.81 (t, 2 H, $CH_2C_6H_5$), 3.34 (t, 2 H, CH_2 Thiox.), 7.16 – 7.59 (m, 11 H, $H_{ar.}$), 8.47 (dd, 1 H, 8-$H_{TX.}$). – ^{13}C NMR (100 MHz, $CDCl_3$): δ = 33.4 (-, CH_2, $CH_2CH_2CH_2$), 36.2 (-, CH_2, CH_{2TX}), 36.5 (-, CH_2, CH_{2Ph}), 124.6 (+, CH, 4-CH_{TX}), 125.3 (+, CH, 4’ *C*H), 125.8 (+, CH, 7-CH_{TX}), 126.2 (+, CH, 8-CH_{TX}), 128.0 (Cq, C_{TX}), 128.4 (+, CH, 2’-*C*H, 6’-*C*H), 128.6 (+, CH, 3’-*C*H, 5’-*C*H), 129.8 (+, CH, 5-CH_{TX}), 129.9 (+, CH, 2-CH_{TX}), 131.3 (+,CH, 6-CH_{TX}), 131.7 (Cq, C_{TX}), 131.8 (Cq, C_{TX}), 135.0 (Cq, C_{TX}), 138.8 (Cq, C_{TX}), 142.7 (Cq, C_{TX}), 148.1 (Cq, C_{Ph}), 182.5 (Cq, *C*O). MS (70 eV, EI), *m/z* (%): 330 (35) [M^+], 239 (100) [$C_{15}H_{11}OS^+$], 226 (95) [$C_{14}H_9OS^+$]. – HRMS ($C_{22}H_{18}OS$): calcd. 330.1073; found 330.1071 – IR (ATR): $\tilde{\upsilon}$ (cm^{-1}) = 3023 (vw) 2922 (w), 2849 (w), 1912 (vw), 1626 (m) (CO), 1584 (m), 1493 (w), 1432 (m); 1344 (vw), 1303 (m), 1182 (w), 1154 (w), 1081 (w), 1034 (w), 957 (vw), 928 (vw), 890 (vw), 873 (w), 806 (vw), 784 (w), 740 (m), 714 (m), 696 (m), 670 (m), 647 (w), 581 (vw), 541 (vw), 521 (vw), 484 (w).

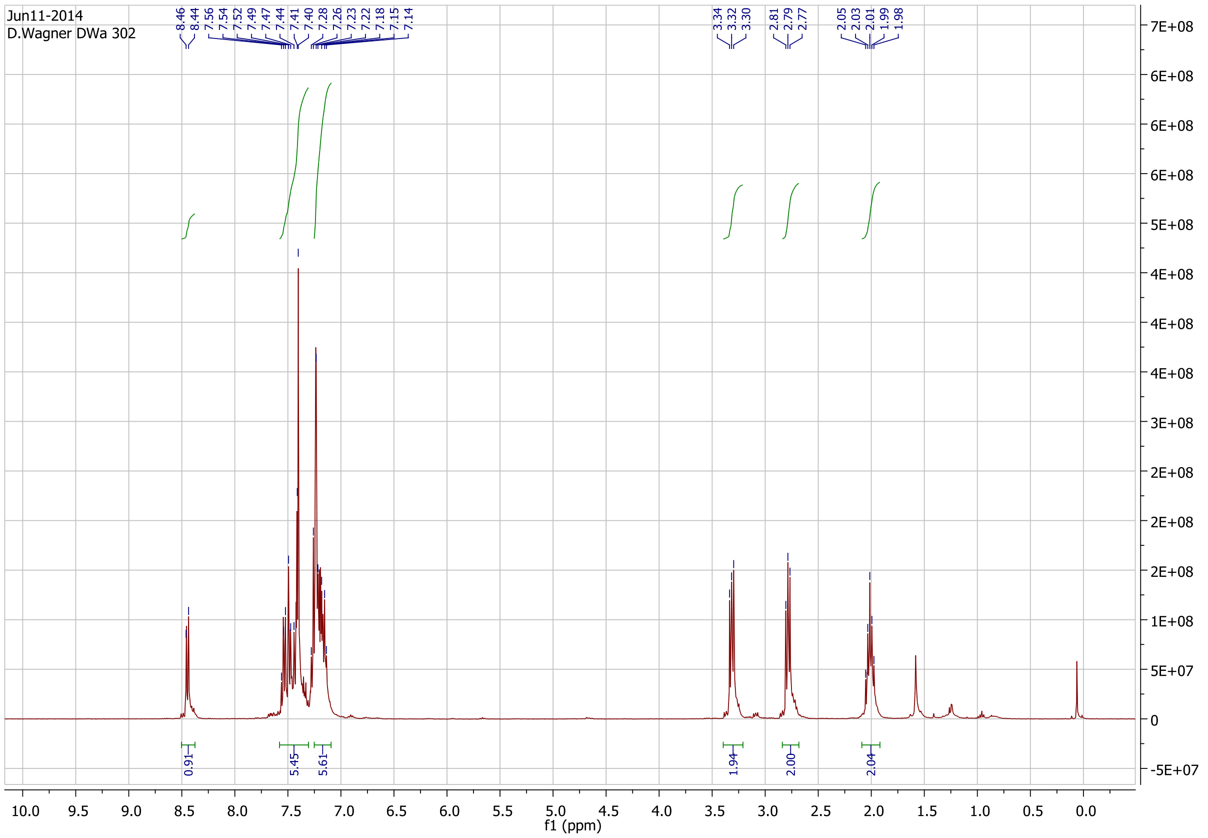
Jun11-2014
D.Wagner DWa 302
8.46
8.44
7.56
7.54
7.52
7.49
7.47
7.44
7.41
7.40
7.28
7.26
7.23
7.22
7.18
7.15
7.14
3.34
3.32
3.30
2.81
2.79
2.77
2.05
2.03
2.01
1.99
1.98
0.91
5.45
5.61
1.94
2.00
2.04
f1 (ppm)

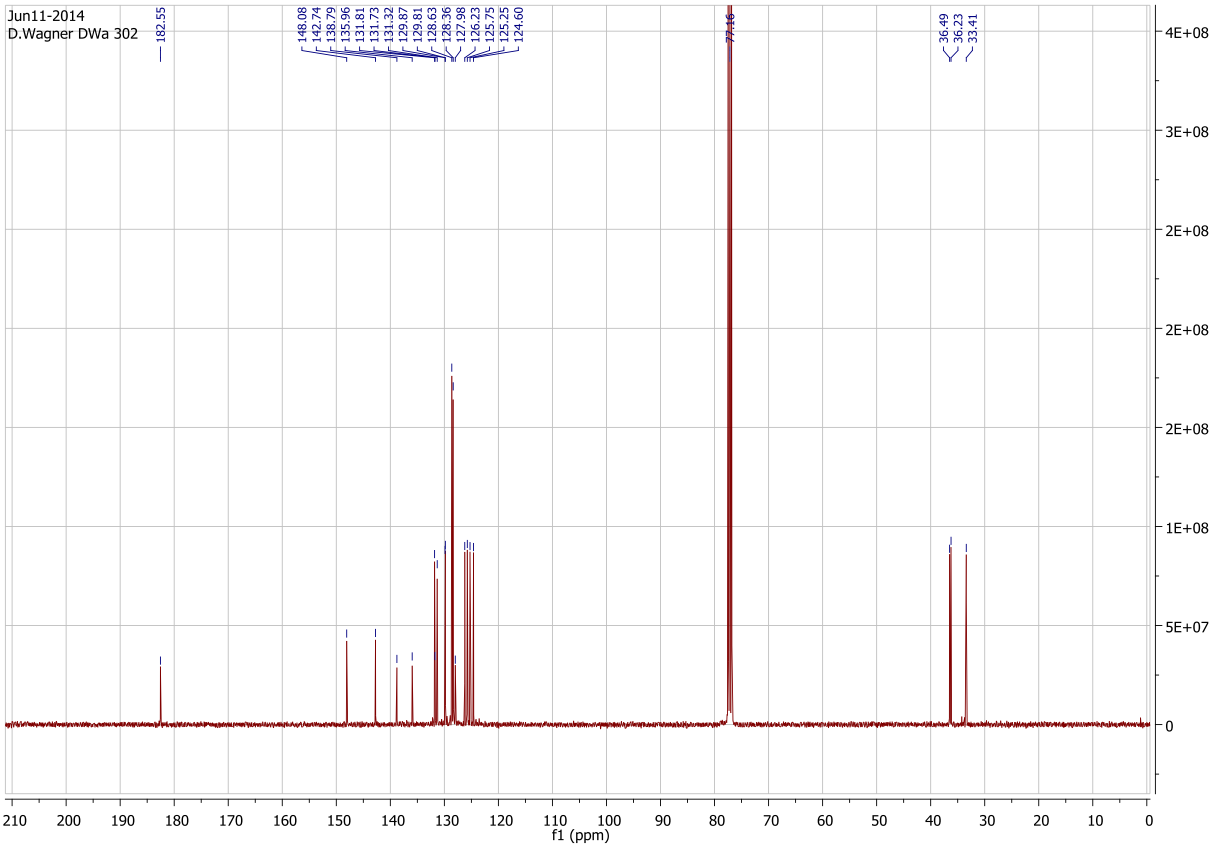
Jun11-2014
D.Wagner DWa 302
182.55
148.08
142.74
138.79
135.96
131.81
131.73
131.32
129.87
129.81
128.63
128.36
127.98
126.23
125.75
125.25
124.60
77.16
36.49
36.23
33.41
f1 (ppm)

1-(3,3-Dimethylbutyl)-9H-thioxanthen-9-one (**1aa**),

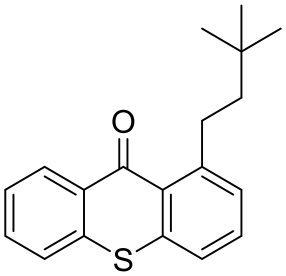

47% yield, clear oil.

^{1}H NMR (400 MHz, $CDCl_3$): δ = 1.05 (s, 9 H, C(CH_3)$_3$), 1.54 (dt, 2 H, CH_2), 3.26 (dt, 2 H, CH_2), 7.23 – 7.26 (dd, 1 H, H_{ar}), 7.39 – 7.45 (m, 3 H, H_{ar}), 7.48 – 7.57 (m, 2 H, H_{ar}), 8.46 (dd, 1 H, H_{ar}) – ^{13}C NMR (100 MHz, $CDCl_3$): δ = 29.6 (+, CH_3, C(CH$_3$)$_3$), 31.0 (C_q , C(CH$_3$)$_3$), 32.0 (-, CH_2, CH$_{2TX}$), 46.5 (-, CH_2, CH$_2$), 124.3 (+, CH, 4-CH$_{TX}$), 125.2 (+, CH, 7-CH$_{TX}$), 126.1 (+, CH, 8-CH$_{TX}$), 128.1 (C_q, C_{TX}), 129.8 (+, CH, 5-CH$_{TX}$), 129.9 (+, CH, 2-CH$_{TX}$), 131.4 (+, CH, 6-CH$_{TX}$), 131.7 (+, CH, 3-CH$_{TX}$), 131.8 (C_q, C_{TX}), 135.9 (C_q, C_{TX}), 138.6 (C_q, C_{TX}), 149.5 (C_q, 1-C_{TX}), 182.5 (C_q, CO). MS (70 eV, EI), *m/z* (%): 296 (35) [M^+], 239 (100) [$C_{15}H_{11}OS^+$]. – HRMS ($C_{19}H_{20}OS$): calcd. 296.1229; found 296.1228. – IR (ATR): $\tilde{\upsilon}$ (cm^{-1}) = 2950 (w), 2859 (w), 1630 (m) (CO), 1583 (m), 1556 (w), 1462 (w), 1443 (m), 1361 (w), 1291 (m), 1235 (w), 1181 (w), 1153 (w), 1126 (w), 1083 (w), 1050 (vw), 1035 (vw), 1009 (vw), 957 (vw), 930 (vw), 872 (w), 811 (vw), 787 (m), 760 (w), 746 (m), 716 (w), 670 (w), 651 (w), 614 (w), 556 (vw). – EA: calcd. for $C_{19}H_{20}OS$: C 76.99, H 6.80, S 10.82; found C 77.12, H 7.07, S 10.42

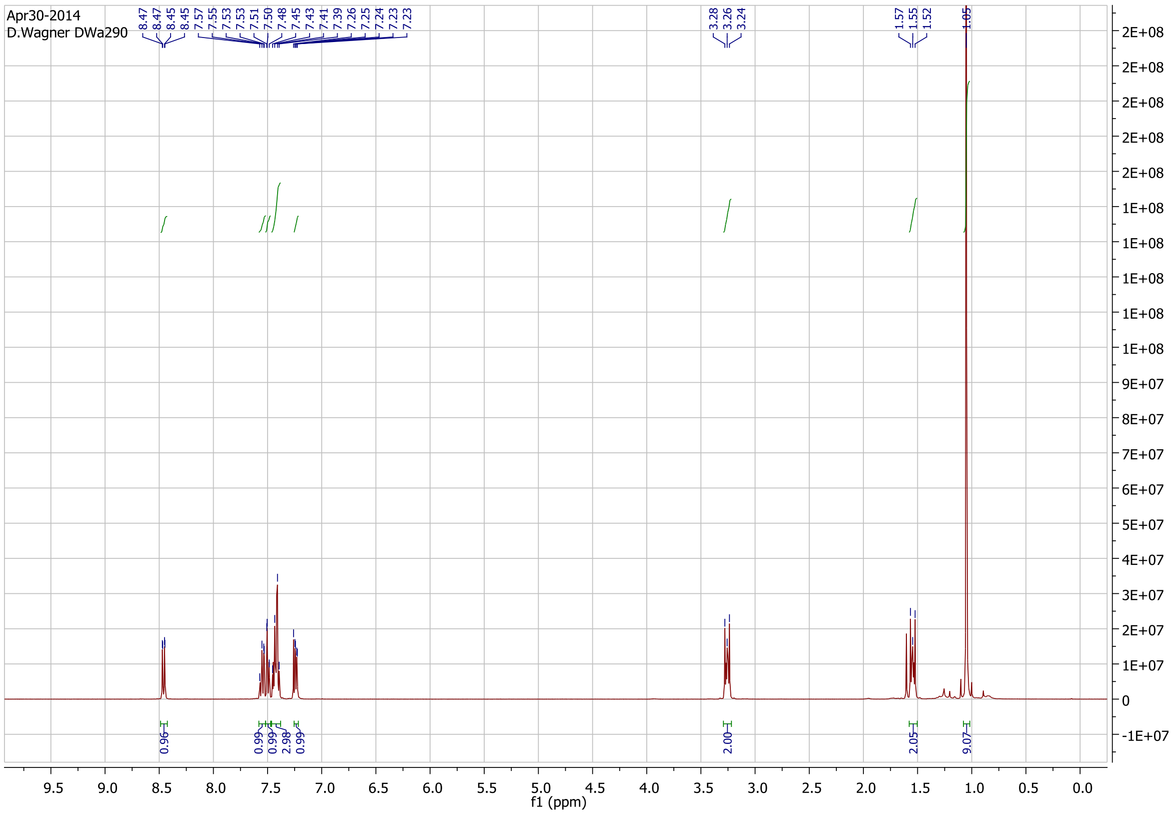

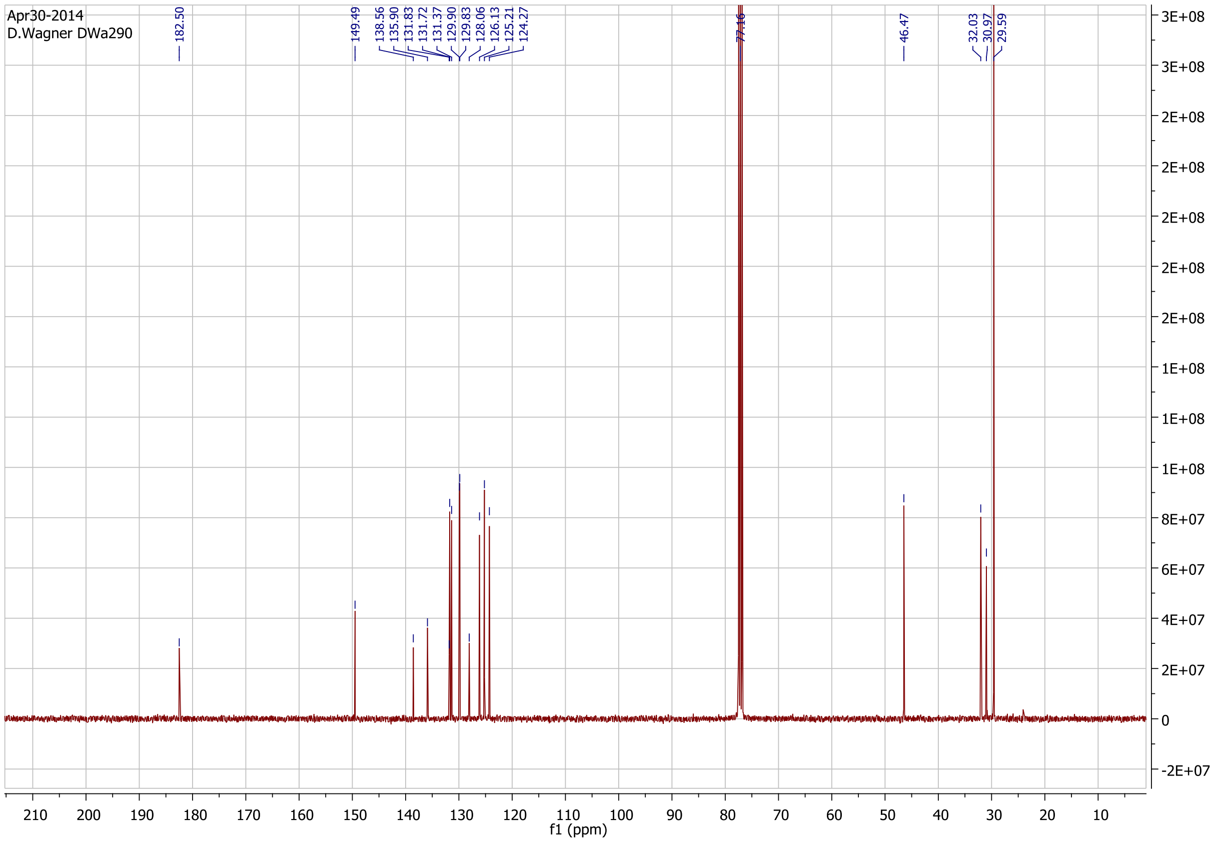

1,8-Bis(3,3-dimethylbutyl)-9H-thioxanthen-9-one (**1aa$_{\mathbf{bis}}$**)

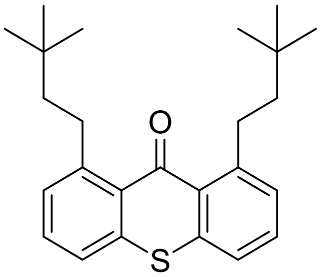

20% yield, clear oil.

^{1}H NMR (400 MHz, $CDCl_3$): δ = 1.02 (s, 18 H, 2 × C(CH_3)$_3$), 1.63 (dt, 4 H, 2 x CH_2), 3.03 (dt, 4 H, 2 x CH_2), 7.20 – 7.22 (dd, 2 H, 2,6-H_{TX}), 7.32 – 7.38 (m, 4 H, 3,4,5,6-H_{TX}). ^{13}C NMR (100 MHz, $CDCl_3$): δ = 29.6 (+, CH_3, C(CH$_3$)$_3$), 30.0 (-, CH_2, CH$_{2TX}$), 31.0 (C_q, C(CH$_3$)$_3$), 46.9 (-, CH_2, CH$_2$), 123.2 (+, CH, 4,5-CH$_{TX}$), 129.1 (+, CH, 2,7-CH$_{TX}$), 130.5 (+, CH, 3,6-CH$_{TX}$), 131.8 (C_q, C_{TX}), 136.4 (C_q, C_{TX}), 146.6 (C_q, C_{TX}), 187.0 (C_q, CO). MS (70 eV, EI), *m/z* (%): 380 (70) [M^+], 323 (100) [$C_{21}H_{23}OS^+$]. – HRMS ($C_{25}H_{32}OS$): calcd. 380.2168; found 380.2167. – IR (ATR): $\tilde{\upsilon}$ (cm^{-1}) = 2943 (vw), 2860 (vw), 1656 (w) (CO), 1577 (vw), 1560 (vw), 1445 (w), 1361 (vw), 1314 (vw), 1284 (w), 1235 (vw), 1181 (vw), 1151 (vw), 1049 (vw), 918 (vw), 863 (vw), 814 (vw), 779 (w), 750 (vw), 686 (vw), 601 (vw), 552 (vw), 450 (vw). EA: calcd. for $C_{25}H_{32}OS$: C 78.90, H 8.48, S 8.42; found C 78.91, H 8.68, S 8.12

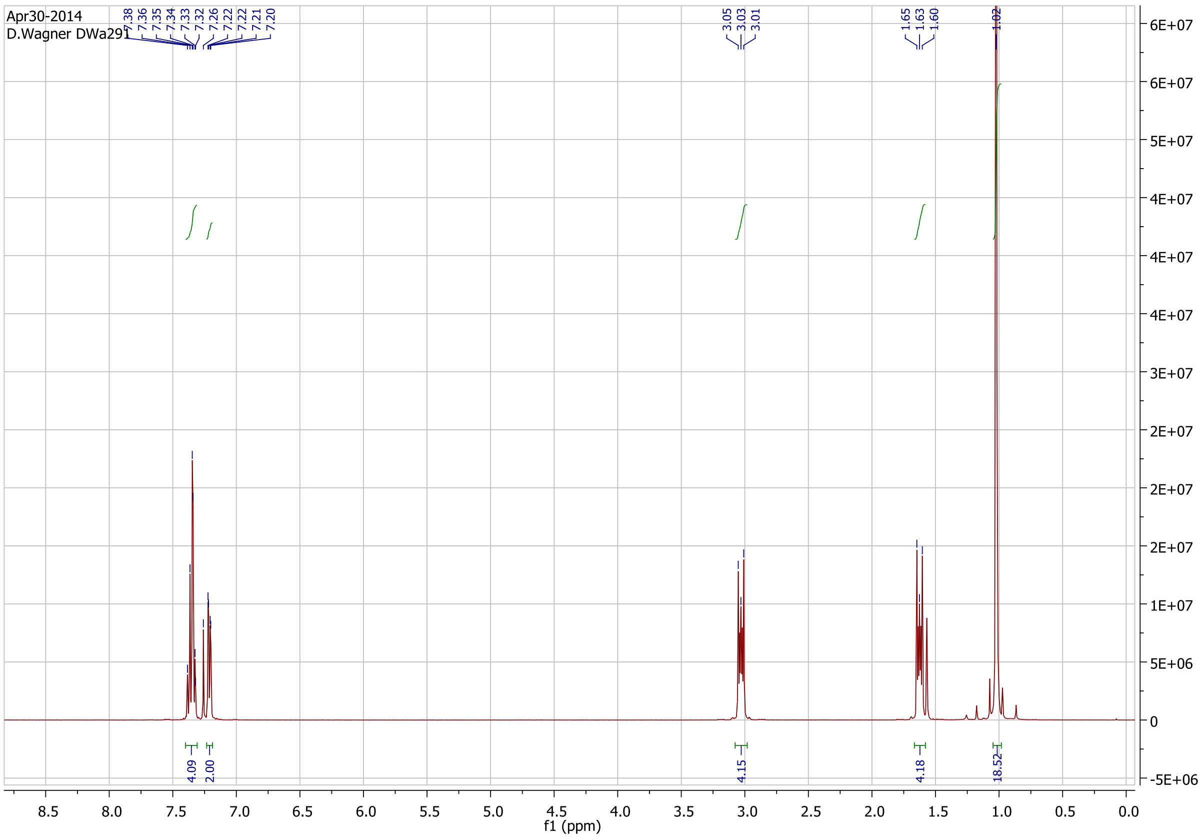
Apr30-2014
D.Wagner DWa291
7.38 7.36 7.35 7.34 7.33 7.32 7.26 7.22 7.22 7.21 7.20
3.05 3.03 3.01
1.65 1.63 1.60
1.02
4.09 2.00 4.15 4.18 18.52
f1 (ppm)

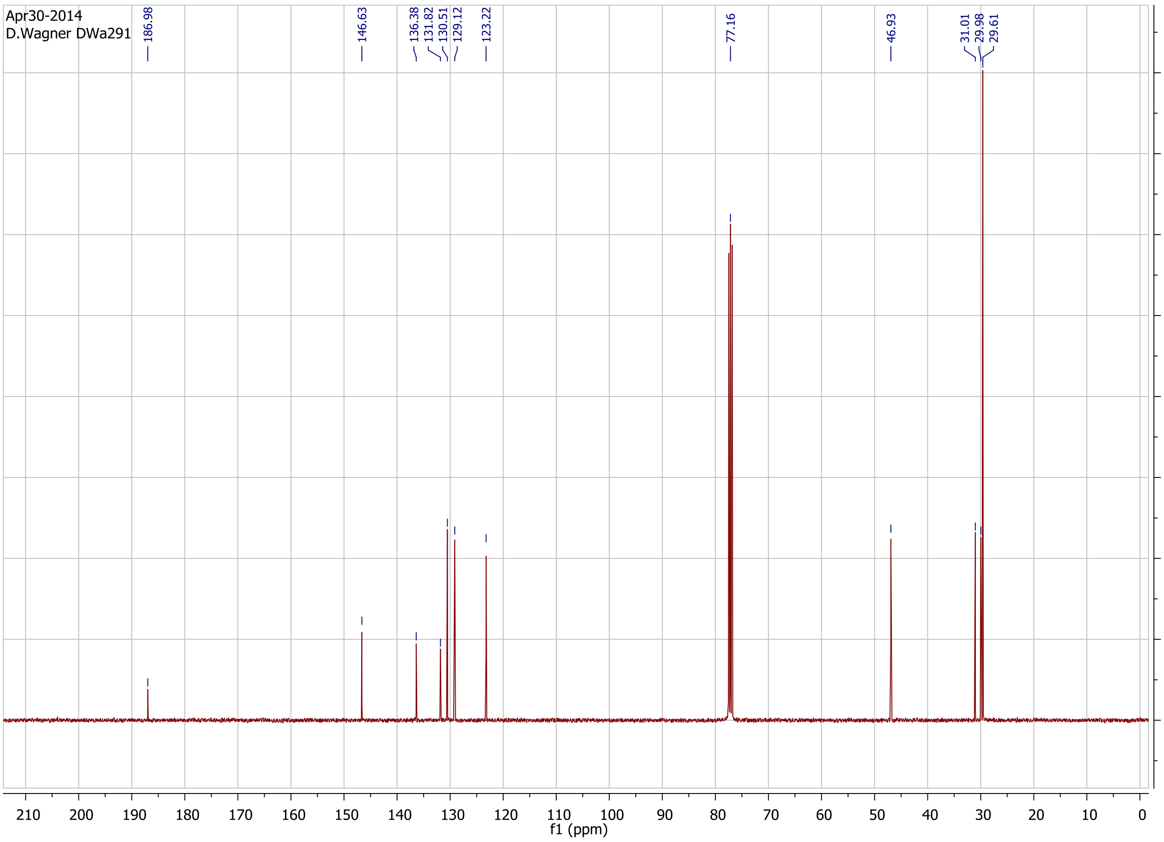
Apr30-2014
D.Wagner DWa291
186.98
146.63
136.38 131.82 130.51 129.12
123.22
77.16
46.93
31.01 29.98 29.61
f1 (ppm)

1,8-Dihexyl-9H-thioxanthen-9-one (**1ab$_{bis}$**)

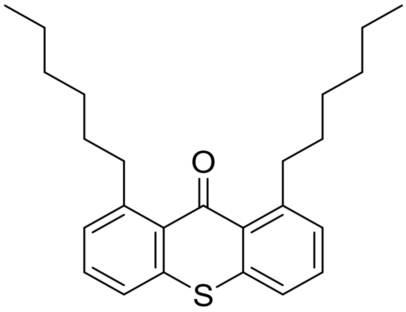

43% yield, clear oil.

^{1}H NMR (400 MHz, $CDCl_3$): δ = 0.90 (t, 6 H, 2 x CH_3), 1.33 – 1.43 (m, 12 H, 2 x CH_2CH_2C$H_2$$CH_3$), 1.67 (m, 2 H, C_4H_9C$H_2$$CH_2$), 3.09 (dt, 2 H, C_5H_{11}CH_2), 7.21 – 7.24 (m, 2 H, 2,7-CH_{TX}), 7.33 – 7.36 (m, 4 H, 3,4,5,6-C_{TX}). ^{13}C NMR (100 MHz, $CDCl_3$): δ = 14.3 (+, CH_3, *C*H_3), 22.9 (-, CH_2, CH_3*C*H_2), 29.8 (-, CH_2, C_2H_5*C*H_2), 31.9 (-, CH_2, C_3H_7*C*H_2), 32.4 (-, CH_2, C_4H_9*C*H_2), 34.4 (-, CH_2, C_5H_{11}*C*H_2), 123.4 (+, CH, 4-*C*H_{TX}), 128.9 (+, CH, 2-*C*H), 130.4 (+, CH, 3-*C*H), 131.8 (C_q, C_{TX}), 136.5 (C_q, C_{TX}), 145.7 (C_q, C_{TX}), 187.5 (C_q, *C*O).). – MS (70 eV, EI), *m/z* (%): 380 (100) [M^+]. – HRMS ($C_{25}H_{32}OS$): calcd. 380.2168; found 380.2168. – IR (ATR): $\tilde{\upsilon}$ (cm^{-1}) = 3444 (vw), 3055 (vw), 2956 (w), 2926 (w), 2855 (w), 1653 (w) (CO), 1581 (w), 1560 (vw), 1445 (w), 1378 (vw), 1288 (vw), 1174 (vw), 1118 (vw), 861 (vw), 779 (w), 722 (vw), 687 (vw), 542 (vw). – EA: calcd. for $C_{25}H_{32}OS$: C 78.90, H 8.48, S 8.42; found C 79.40, H 8.09, S 7.67.

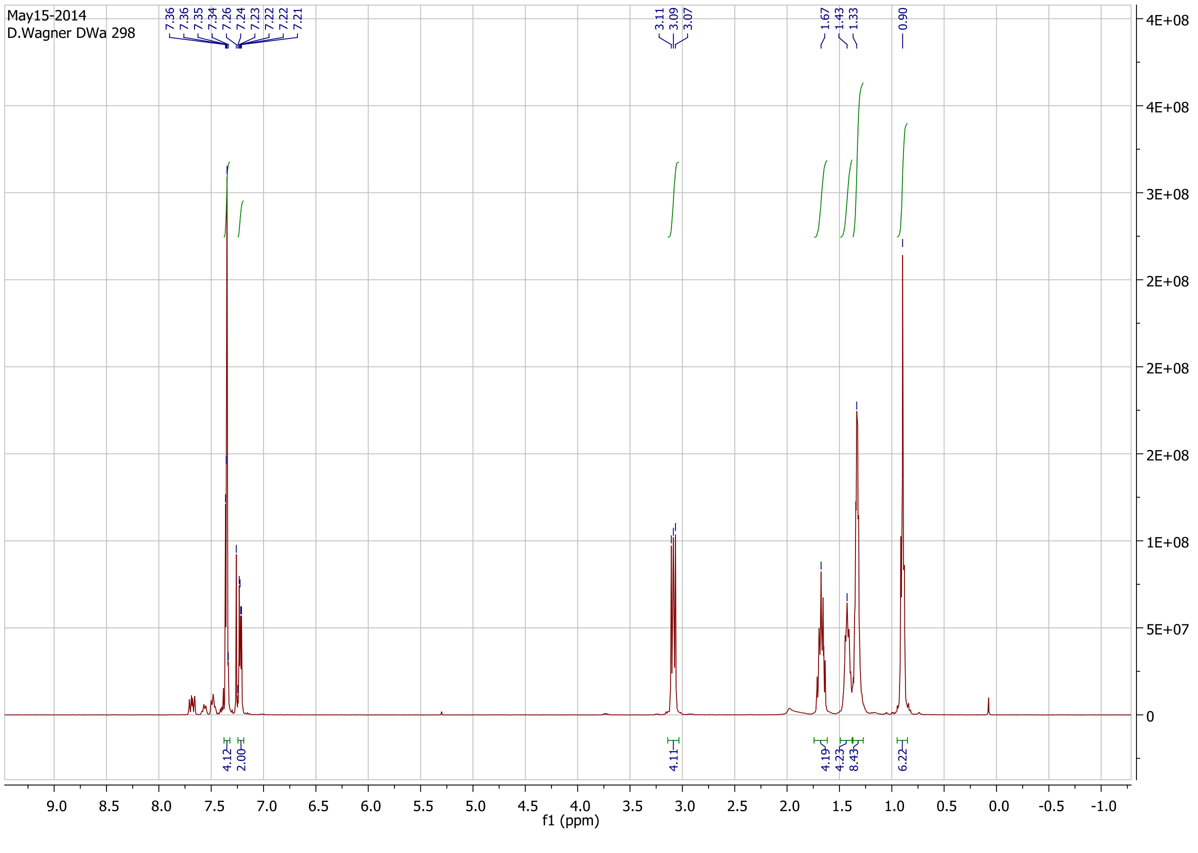

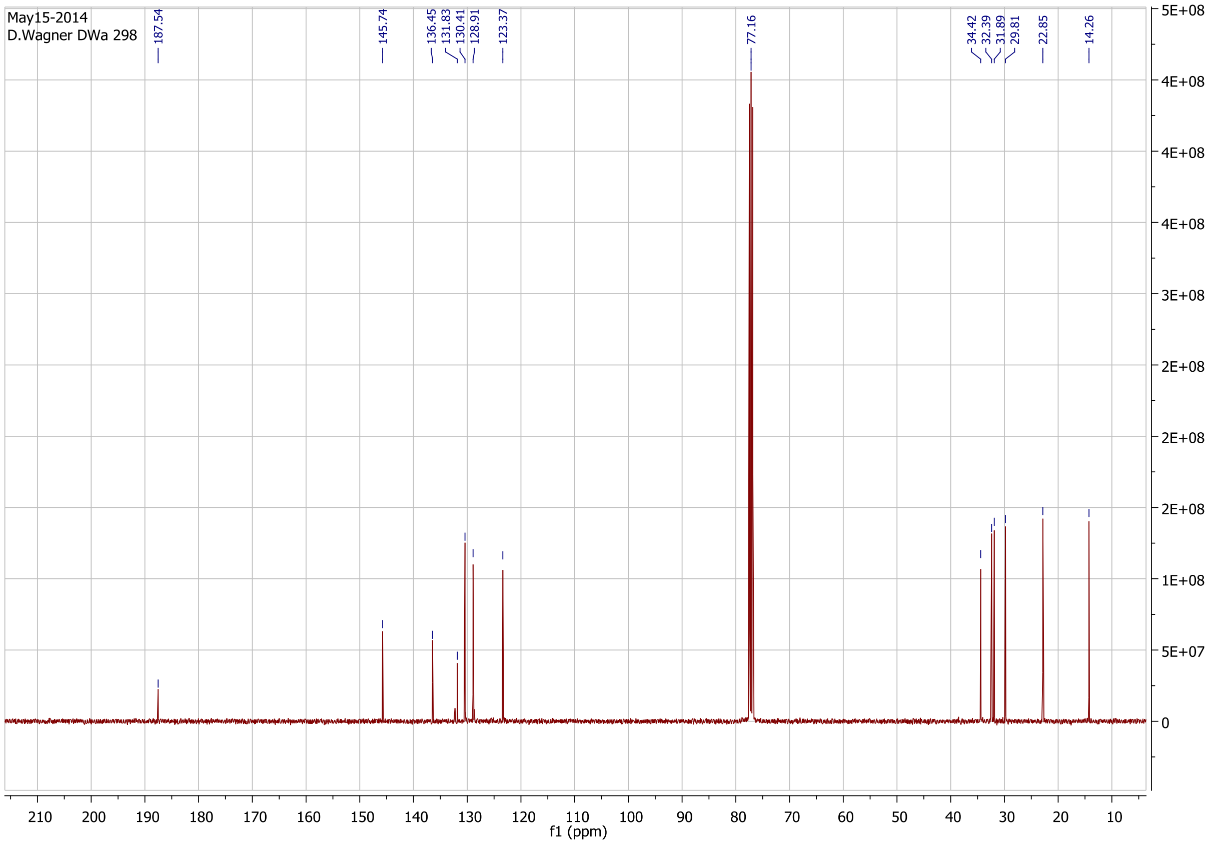

8-(3,3-Dimethylbutyl)-4-methoxy-1-methyl-9H-thioxanthen-9-one (**1ha**)

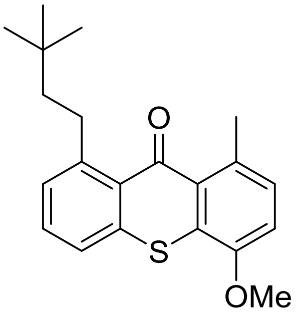

83%, clear oil

^{1}H NMR (400 MHz, $CDCl_3$): δ = 1.01 (s, 9 H, C(CH_3)$_3$), 1.51 (dt, 2 H, CH_2), 2.67 (s, 3 H, CH_3), 3.13 (dt, 2 H, CH_2), 3.98 (s, 3 H, OCH_3), 6.92 (d, 1 H, $H_{ar.}$), 7.15 – 7.22 (m, 2 H, $H_{ar.}$), 7.34 -7.42 (m, 2 H, $H_{ar.}$) –^{13}C NMR (100 MHz, $CDCl_3$): δ = 21.5 (+, CH_3 Thiox.), 29.4 (+, C(CH)$_3$), 30.1 (-, CH_2, CH_2), 31.0 ($C_{q.}$, C(CH_3)$_3$), 46.9 (-, CH_2, CH_2), 56.6 (+, CH_3, OCH_3), 111.2 (+, CH, 3-CH_{TX}), 124.1 (+, CH, 5-CH_{TX}), 126.2 (C_q, C_{TX}), 129.1 (+, CH, 2-CH_{TX}), 129.2 (+, CH, 7-CH_{TX}), 130.6 (+, CH, 6-CH_{TX}), 131.2 ($C_{q.}$, C_{TX}), 132.2 ($C_{q.}$, C_{TX}), 132.3 ($C_{q.}$, C_{TX}), 136.9 ($C_{q.}$, C_{TX}), 146.6 ($C_{q.}$, C_{TX}), 152.2 ($C_{q.}$, C_{TX}) ,187.2 ($C_{q.}$, CO). ppm. MS (70 eV, EI), *m/z* (%): 283 (100) [$C_{17}H_{15}O_2S^+$], 340 (50) [M^+]. – HRMS ($C_{21}H_{24}O_2S$): calcd. 340.1492; found 340.1491. – IR (ATR): $\tilde{\upsilon}$ (cm^{-1}) = 2954 (w), 1646 (w), 1560 (w), 1444 (w), 1392 (w), 1363 (vw), 1293 (w), 1259 (w), 1244 (w), 1216 (w), 1183 (w), 1063 (w), 954 (w), 859 (w), 813 (w), 802 (w), 786 (w), 752 (vw), 724 (vw), 689 (w), 634 (vw), 617 (vw), 565 (vw), 547 (vw), 442 (vw). EA: calcd. for $C_{21}H_{24}O_2S$: C 74.08, H 7.11, S 9.42; found C 74.29, H 7.19, S 9.33

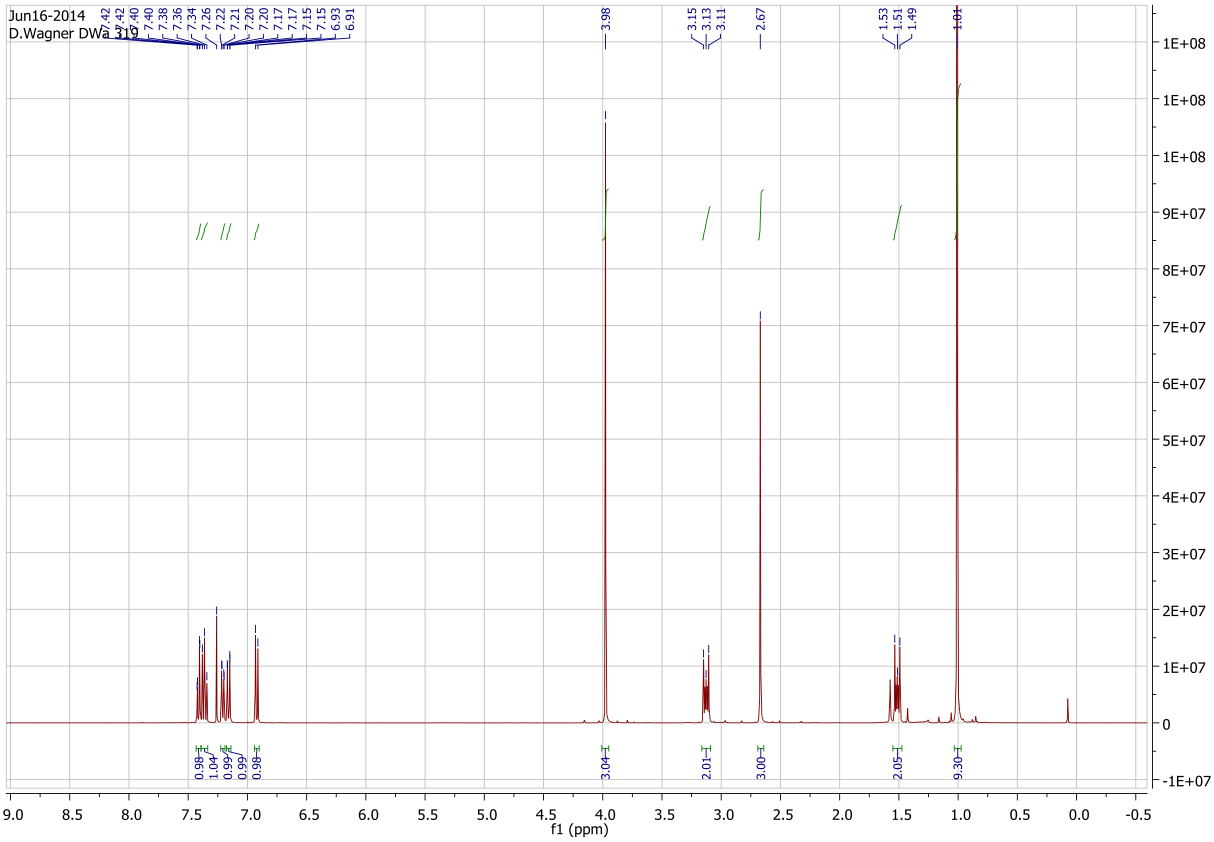
Jun16-2014
D.Wagner DWa 319
7.42
7.42
7.40
7.40
7.38
7.36
7.34
7.26
7.22
7.21
7.20
7.20
7.17
7.17
7.15
7.15
6.93
6.91
3.98
3.15
3.13
3.11
2.67
1.53
1.51
1.49
1.01
0.98
1.04
0.99
0.99
0.98
3.04
2.01
3.00
2.05
9.30
f1 (ppm)

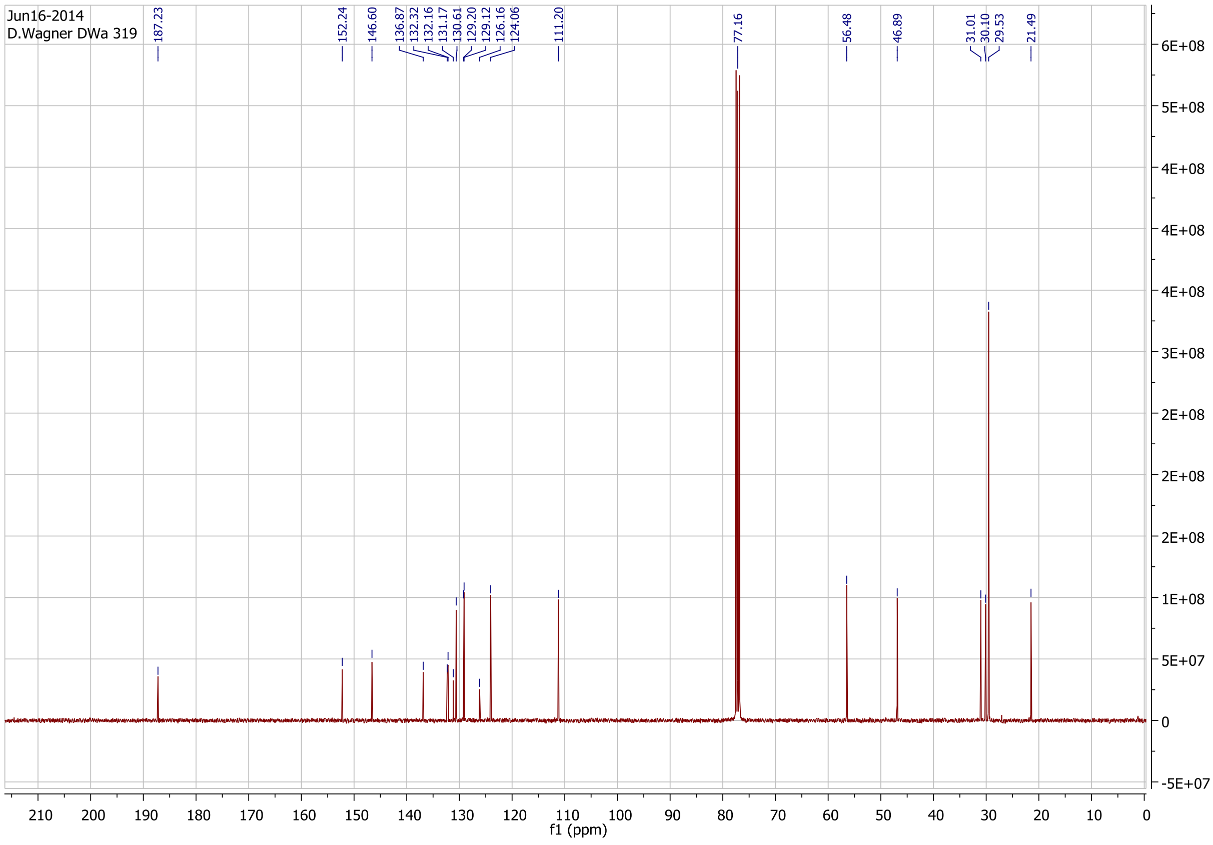
Jun16-2014
D.Wagner DWa 319
187.23
152.24
146.60
136.87
132.32
132.16
131.17
130.61
129.20
129.12
126.16
124.06
111.20
77.16
56.48
46.89
31.01
30.10
29.53
21.49
f1 (ppm)

8-Hexyl-4-methoxy-1-methyl-9H-thioxanthen-9-one (**1hb**)

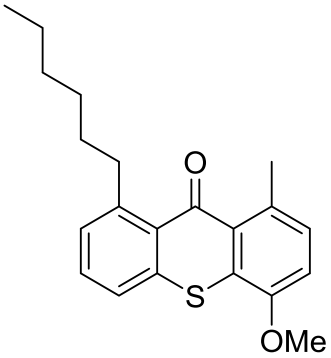

65%, clear oil.

^{1}H NMR (400 MHz, $CDCl_3$): δ = 0.88 (t, 3 H, CH_2CH_3), 1.29-1.40 (m, 6 H, $CH_2CH_2CH_2$), 1.64 (m, 2 H, CH_2), 2.66 (s, 3 H, CH_3 Thiox.), 3.14 (t, 2 H, CH_2 Thiox.), 3.98 (s, 3 H, OCH_3), 6.92 (d, 1 H, $H_{ar.}$), 7.15 – 7.22 (m, 2 H, $H_{ar.}$), 7.35 -7.43 (m, 2 H, $H_{ar.}$) – ^{13}C NMR (100 MHz, $CDCl_3$): δ = 14.2 (+, CH_3 Hex.), 21.5 (+, CH_3 Thiox.), 22.8 (-, CH_2, Hex.), 29.7 (-, CH_2, Hex.), 31.9 (-, CH_2, Hex.), 32.3 (-, CH_2, Hex.), 34.7 (-, CH_2, Hex.), 56.5 (+, CH_3, OCH_3), 111.2 (+, CH, 5-CH_{TX}), 124.2 (+, CH, 2-CH_{TX}), 126.2 (C_q, C_{TX}), 129.0 (+, CH, 7-CH_{TX}), 129.1 (+, CH, 3-CH_{TX}), 130.5 (+, CH, 3-CH_{TX}), 131.1 ($C_{q.,}$ C_{TX}), 132.2 ($C_{q.,}$ C_{TX}), 132.3 ($C_{q.,}$ C_{TX}), 136.9 ($C_{q.,}$ C_{TX}), 145.9 ($C_{q.,}$ C_{TX}), 152.2 ($C_{q.,}$ C_{TX}), 187.3 ($C_{q.,}$ CO_{TX}) ppm. – MS (70 eV, EI), *m/z* (%): 340 (100) [M+], 283 (75) [$C_{17}H_{15}O_2S$+]. – HRMS ($C_{21}H_{24}O_2S$): calcd. 340.1492; found 340.1493 – IR (ATR): $\tilde{\nu}$ (cm^{-1}) = 2923 (w), 2853 (w), 1642 (m), 1573 (w), 1560 (w), 1433 (m), 1380 (vw), 1286 (w), 1257 (m), 1215 (m), 1064 (m), 958 (w), 858 (w), 801 (w), 780 (w), 725 (w), 692 (w), 570 (vw), 437 (vw). – EA: calcd. for $C_{21}H_{24}O_2S$: C 74.08, H 7.11, S 9.42; found C 74.65, H 7.27, S 9.44

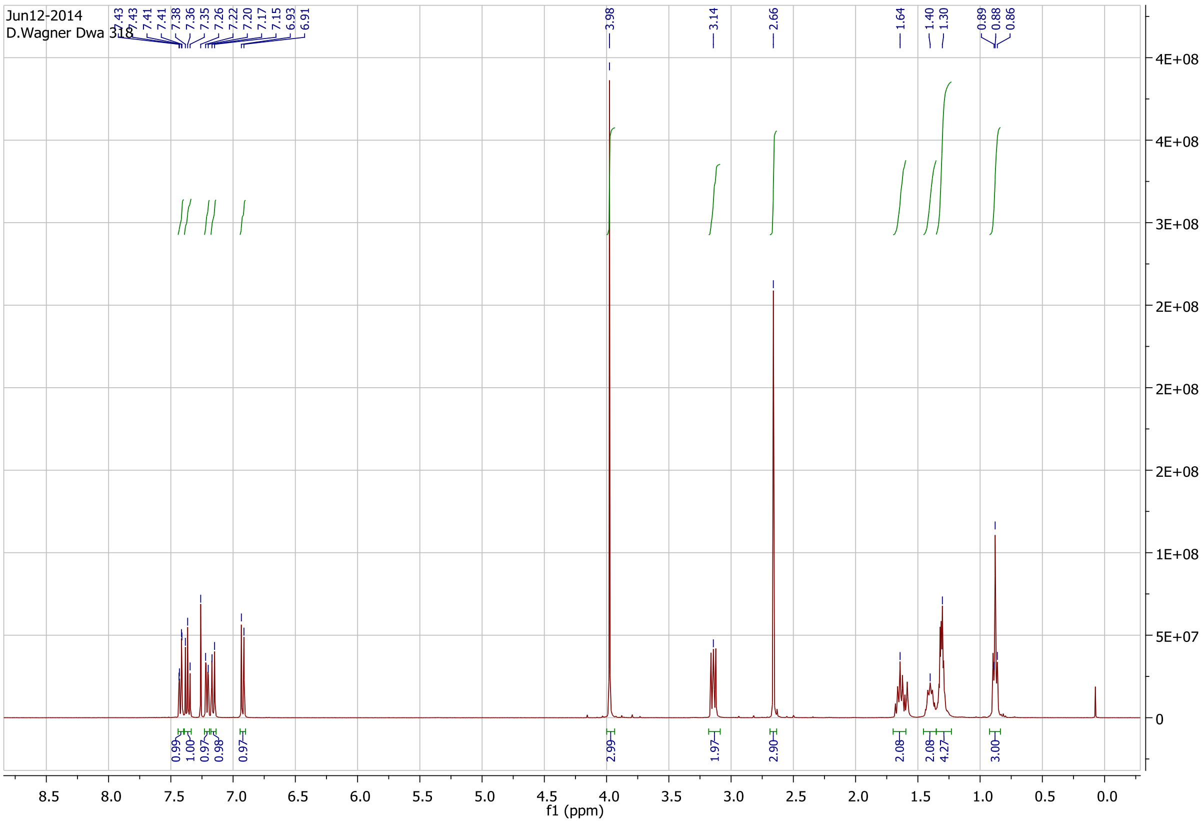

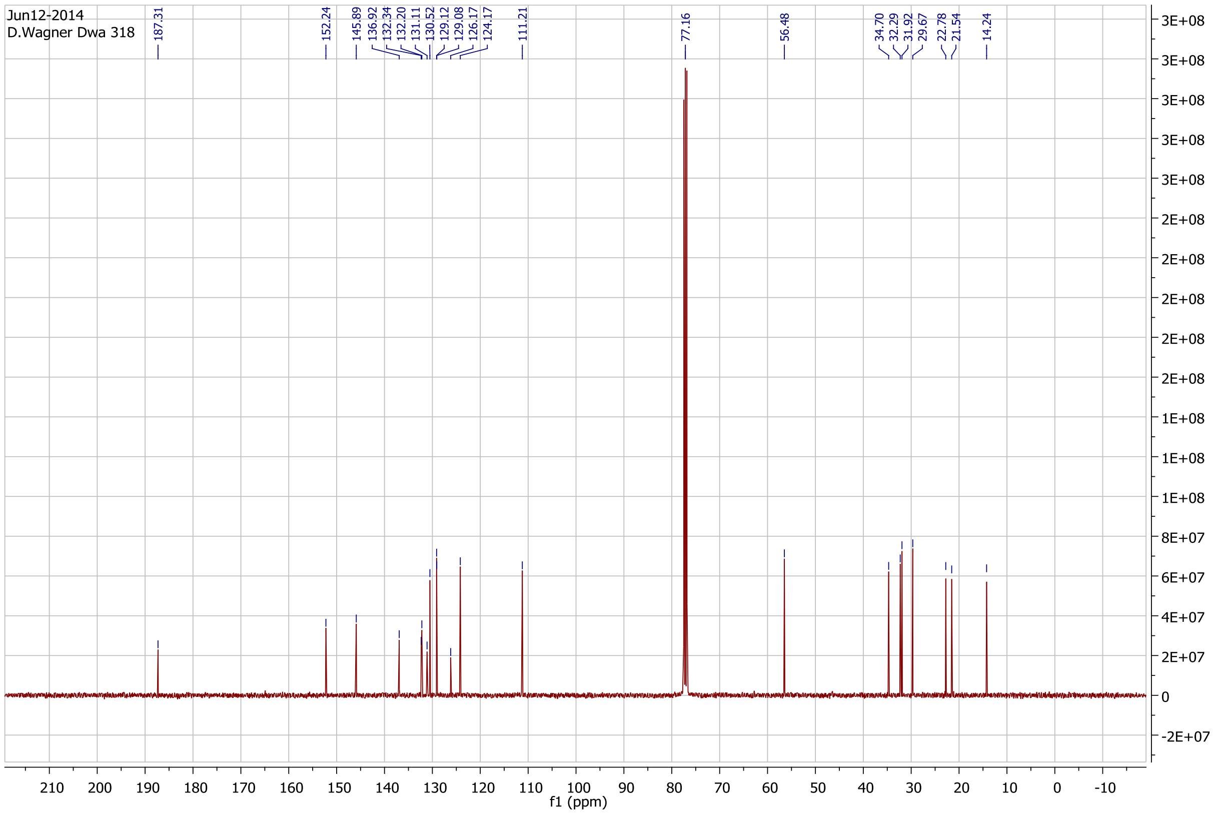

Jun12-2014
D.Wagner Dwa 318
187.31
152.24
145.89
136.92
132.34
132.20
131.11
130.52
129.12
129.08
126.17
124.17
111.21
77.16
56.48
34.70
32.29
31.92
29.67
22.78
21.54
14.24
f1 (ppm)
